# Reverse vaccinology approaches to design a potent multiepitope vaccine against the *HIV* whole genome: immunoinformatic, bioinformatics, and molecular dynamics approaches

**DOI:** 10.1186/s12879-024-09775-2

**Published:** 2024-08-28

**Authors:** Ava Hashempour, Nastaran Khodadad, Shokufeh Akbarinia, Farzane Ghasabi, Younes Ghasemi, Mohamad Matin Karbalaei Ali Nazar, Shahab Falahi

**Affiliations:** 1 HIV/AIDS Research Center, Institute of Health, Shiraz University of Medical Sciences, Shiraz, Iran; 2 Pharmaceutical Sciences Research Center, Shiraz University of Medical Sciences, Shiraz, Iran; 3 Department of Biotechnology, School of Pharmacy, Shiraz University of Medical Sciences, Shiraz, Iran; 4 Zoonotic Diseases Research Center, Ilam University of Medical Sciences, Ilam, Iran

**Keywords:** *HIV*, Vaccine, Bioinformatic, TLR, Molecular dynamic, Main *HIV* subtypes and CRF

## Abstract

Substantial advances have been made in the development of promising *HIV* vaccines to eliminate HIV-1 infection. For the first time, one hundred of the most submitted HIV subtypes and CRFs were retrieved from the LANL database, and the consensus sequences of the eleven HIV proteins were obtained to design vaccines for human and mouse hosts. By using various servers and filters, highly qualified B-cell epitopes, as well as HTL and CD8 + epitopes that were common between mouse and human alleles and were also located in the conserved domains of *HIV* proteins, were considered in the vaccine constructs. With 90% coverage worldwide, the human vaccine model covers a diverse allelic population, making it widely available. Codon optimization and *in silico* cloning in prokaryotic and eukaryotic vectors guarantee high expression of the vaccine models in human and *E. coli* hosts. Molecular dynamics confirmed the stable interaction of the vaccine constructs with TLR3, TLR4, and TLR9, leading to a substantial immunogenic response to the designed vaccine. Vaccine models effectively target the humoral and cellular immune systems in humans and mice; however, experimental validation is needed to confirm these findings *in silico*.

**Supplementary Information:**

The online version contains supplementary material available at 10.1186/s12879-024-09775-2.

## Introduction

Mortality from pathogenic agents has decreased globally; nevertheless, infectious diseases still inflict great catastrophes in human history [1–6]. For example, acquired immunodeficiency syndrome (AIDS) is a global health crisis caused by human immunodeficiency virus (*HIV*)-1 and is challenging to diagnose and treat [7]. Two types of *HIV*, *HIV-1* and *HIV-2*, are recognized as the major causes of the AIDS pandemic, and *HIV-1* is the most prevalent type [8, 9].

Antiretroviral therapy (ART) prevents the spread of *HIV* infection, slows the progression of AIDS, and extends the lifespan; hence, ART cannot eliminate *HIV* in the human body [7]. Therefore, there are still numerous obstacles to controlling or eradicating *HIV* infection that necessitate the development of an efficient vaccine. The global burden of *HIV*-infected individuals has been reduced significantly by the introduction of effective drugs such as ritonavir, dolutegravir, and efavirenz [10]. However, it is believed that a successful vaccine is needed to eradicate *HIV* [11].

There are several new approaches for developing an efficient *HIV* vaccine. Nonetheless, the main difficulties preventing the production of an optimal vaccine include the following: (1) *HIV* replication introduces a high rate of mutation in its genome that leads to the creation of new subtypes and circulating recombinant forms (CRFs) and strains; (2) *HIV-1* establishes a latent reservoir; (3) there is no suitable animal model for investigating *HIV* behavior; (4) there are several funding issues associated with vaccine development [12]; (5) the designed vaccines are unable to induce both helper T-cell and cellular responses [13] and fail to produce broadly neutralizing antibodies; and (6) there are a wide variety of ways in which *HIV* can be transmitted to humans [14]. This is why the *HIV* vaccine cannot induce a sufficient immune response in the worldwide human population; for example, one of the most efficiently designed vaccines was RV144, which provides only 31.2% protection against *HIV* [15].

Different forms of vaccination are used to eradicate or control *HIV* infections, among which the conventional method is a very old approach that involves the use of live attenuated or dead organisms [16]. Currently, analyzing immunological data is the most efficient and powerful strategy for developing vaccines [17]. For example, multiepitope vaccines for several pathogens have attracted increased amounts of attention worldwide [18]. Furthermore, bioinformatic approaches have several advantages, including success in preclinical models, safety, specificity of the target, simplicity of production, etc. [19].

In this computational study, the vaccine models were well matched to the main *HIV* subtypes and CRFs that targeted the humoral and cellular immune systems in computer-based immune response simulations in both human and mouse hosts. For this purpose, one hundred *HIV-1* sequences of the most prevalent subtypes and CRFs were retrieved from the Los Alamos National Laboratory (LANL) database, and consensus sequences of the main genes [*Gag*, protease (*Pro*), integrase (*IN*), reverse transcriptase (*RT*), and envelope (*Env*)], regulatory genes (*Tat* and *Rev*), and accessory genes (*Vif*, *Nef*, *Vpr*, and *Vpu*)] were generated. Using various servers, thousands of B cell, CD4, and CD8 primary epitopes were predicted and screened for distinctive criteria, including population coverage, antigenicity, allergenicity, toxicity, immunogenicity, topology, homology, and IFN-γ induction. Among several epitopes, only those located in the conserved domains of the two ORFs and nine proteins were candidates in the vaccine construct sequence. The secondary and tertiary structures of the vaccine constructs were investigated, and molecular docking and molecular dynamics studies of Toll-like receptors (TLRs) 3, 4, and 9 confirmed that the vaccine construct would not lose its effectiveness in the long run. Along with suitable adjuvants comprising beta defensin-3, a pan-HLA DR-binding epitope (PADRE), and the C-terminal invasin sequence of Yersinia, the vaccine construct can simulate the innate response through appropriate TLR dockings and induce appropriate proinflammatory cytokines such as IFN-ɣ. Finally, codon optimization and *in silico* cloning of the adeno-based vaccines and pET28(a) plasmid were performed to efficiently express the vaccine constructs in human and mouse host cells.

## Materials and methods

### Data retrieval, sequence alignment, and conservancy analysis

To design effective vaccine candidates against *HIV*, we retrieved the full genomes of the main *HIV* subtypes and CRFs [B (55%), C (15%), A (7%), AE (7%), D (3%), G (3%), F (1%), AG (3%), BC (2%), O (2%), BF (1%), and CRF35-AD (1%)] from the LANL *HIV* sequence database (www.hiv.lanl.gov) on 4th June 2023. This database provides valuable data, namely, the geographic distribution of *HIV-1* sequences and subtypes derived from published reports.

Using CLC-sequence viewer software (version CLC Genomics Workbench 20) [20], the amino acid sequences of two ORFs (*Gag* and *Env*) and nine genes (*Pro*,* IN*,* RT*,* Tat*,* Nef*,* Rev*,* Vif*,* Vpr*, and *Vpu*) were obtained from each *HIV* whole genome. The homology among the sequences was examined by CLC-sequence viewer software with the following parameters: gap extension cost: 1.0, gap opening cost: 10, and very accurate progressive alignment algorithm. This step was followed by the alignment of one hundred sequences of two ORFs and nine genes to generate consensus sequences, which were considered for analysis to design the vaccine construct. To identify the conserved domains of the consensus protein sequences, we used NCBI CDD-BLAST (https://www.ncbi.nlm.nih.gov/Structure/cdd/wrpsb.cgi).

In addition, two distinct datasets, the National Center for Biotechnology Information (NCBI) (https://www.ncbi.nlm.nih.gov/) and the European Nucleotide Archive (ENA) (https://www.ebi.ac.uk/ena/browser/home), were utilized to obtain 100 unique HIV whole genomes from NCBI and ENA that were not duplicated across the three datasets (LANL, NCBI, and ENA). Two new groups of 100 HIV-completed genomes were carefully constructed with similar subtypes, ensuring a comprehensive and representative analysis (Additional Tables 1 and 2). A secondary analysis of all of the other HIV-1 genome sequences was performed to determine how conserved the identified epitopes were representative across the HIV-1 genetic landscape. For this purpose, we downloaded the full genomes of all the subtypes and CRFs that were not included in our study (Additional Table 1) from the HIV LANL. The sequence of each epitope was subsequently compared with the sequences of all the HIV subtypes and CRF sequences.

We derived the consensus sequence from two ORFs and nine genes and identified their conserved domains. The final epitopes selected from the HIV whole genome obtained from the LANL were assessed in the two new consensus sequences generated from the NCBI and ENA databases.

### Prediction of biophysical and biochemical features

The ProtParam tool (https://web.expasy.org/protparam/) [21, 22] was applied to determine the physicochemical properties of two ORFs and nine proteins. The number of amino acids, estimated half-life, molecular weight, theoretical isoelectric point (pI), aliphatic index, instability index, and grand average of hydropathicity (GRAVY) index were analyzed. The pKa of each amino acid present in the protein sequence determines its physiochemical properties [23].

### Prediction and selection of linear B-cell epitopes

To increase the accuracy of B-cell epitope prediction, which accounts for specific humoral immune response stimulation, five methods were used, including the ABCpred server (https://webs.iiitd.edu.in/raghava/abcpred/ABC_submission.html) and four tools in the immune epitope database and analysis resource (IEDB) (http://tools.iedb.org/bcell/), comprising Bepipred V2, the Emini Surface Accessibility Prediction tool, the Karplus flexibility tool, and the Parker Hydrophilicity Prediction method.

The ABCpred server relies on an artificial neural network of 700 B-cell epitopes and 700 non-B-cell epitopes spanning up to 20 residues for training and testing purposes [24–26]. BepiPred was the second server, with a threshold of 1 [27], to analyze the data by integrating various parameters, including physicochemical properties, antigenicity, hydrophilicity, etc. [28]. The third server was the Emini Surface Accessibility tool, which determines which parts of a protein are likely to be exposed on the surface [29]. The fourth and fifth servers are the Karplus flexibility prediction method and the Parker method, which are commonly used for prediction and include flexibility and hydrophilicity, respectively [26]. The suggested B-cell epitopes were screened through the following filters: (1) the VaxiJen server (http://www.ddg-pharmfac.net/vaxijen/) [30, 31] to assess the antigenic score, and epitopes that could not meet the server threshold level (0.4) were discarded; (2) the AllerTOP v.2.0 server (http://www.ddg-pharmfac.net/AllerTOP/) was used to estimate the allergenicity of the selected epitopes [32, 33]; (3) the ToxinPred webserver (https://webs.iiitd.edu.in/raghava/toxinpred/multi_submit.php) was used to characterize the toxicity of the epitopes [34, 35]; (4) the TMHMM server (https://dtu.biolib.com/DeepTMHMM) determined the transmembrane topology of the epitopes [36, 37]; and (5) the PIR peptide matching program (https://research.bioinformatics.udel.edu/peptidematch/index.jsp) [38–40] selected the epitopes that were homologous to HIV genes.

### Prediction and selection of the CD4 helper T lymphocyte (HTL) epitope

The IEDB histocompatibility complex (MHC) II binding server (http://tools.iedb.org/mhcii) applied the NetMHCIIpan-4.1 algorithm that utilizes artificial neural networks (ANNs) to predict peptide binding to known sequence MHC II molecules. Through training on a vast dataset with over 500,000 measurements of binding affinity (BA) and eluted ligand mass spectrometry (EL) data encompassing HLA-DR, HLA-DQ, HLA-DP human MHC class II isotypes, and mouse molecules (H-2), the coverage of the server has been extended. In our study, we inputted a single protein sequence in FASTA format to predict the epitopes via the NetMHCIIpan-4.1 algorithm to identify specific species/loci. If relevant, the α and β chains were chosen separately (H2-IAb, H2-IAd, H2-IEd), along with the selection of a 15-mer length, and then all percentile ranks were set. We subsequently selected all options with a percentile rank below 10 after submission at the end of the use of these alleles for screening [41, 42]. The VaxiJen v2.0, Immunogenicity (https://tools.iedb.org/immunogenicity/), AllerTOP v.2.0, ToxinPred, TMHMM-2.0, topology, and PIR peptide matching programs were subsequently utilized for filtration. Moreover, the qualified epitopes were screened through the IFNepitope webserver (http://crdd.osdd.net/raghava/ifnepitope/predict.php). This server is used to identify interferon-gamma (IFN-γ)-inducing epitopes. The selected epitopes were considered for cross-checking with the IEDB class II immunogenicity server for human leukocyte antigen (HLA) class II binding [43, 44]. Finally, epitopes with the most HLA class-II coverage were subjected to population coverage.

### Prediction and selection of the CD8 + T-cell (CTL) epitope

The consensus sequences of two ORFs and nine proteins were subjected to the NetCTL 1.2 server (https://services.healthtech.dtu.dk/services/NetCTL-1.2/) with a threshold value of 0.5 and other default parameters, including a weight on TAP transport efficiency of 0.05 and a weight on C-terminal cleavage of 0.15, to obtain 9-mer CTL epitopes against HLA class I allele subgroups of 12 superfamilies (A1, A2, A3, A24, A26, B7, B8, B27, B39, B44, B58, and B62) [45, 46]. The epitopes that exhibited good binding affinity with human supertypes were screened through different filters: VaxiJen, AllerTOP, ToxinPred, TMHMM-2.0, and the PIR peptide matching program. The selected epitopes were cross-checked with two servers: the NetMHC 4 server (https://services.healthtech.dtu.dk/services/NetMHC-4.0/) for mouse alleles (H-2-Db, H-2-Dd, H-2-Kb, H-2-Kd, H-2-Kk, and H-2-Ld) [47] and the IEDB class I immunogenicity server for human HLA class-I binding (http://tools.immuneepitope.org/mhci/) [43]. The MHC-I binding prediction tool generates a percentile rank for each epitope‒allele complex to indicate affinity. A lower percentile rank indicates a stronger binding prediction, so a base value of 10 was set to evaluate binding to all MHC class I alleles [48, 49]. Finally, most HLA-containing epitopes were subjected to population coverage analysis, which was included in the human vaccine model.

### Population coverage

The frequency of HLA alleles varies significantly across diverse ethnic groups. Hence, when designing and creating T-cell epitope-based diagnostics or vaccines, it is crucial to choose various epitopes that possess diverse HLA-binding specificities. This approach will result in enhanced coverage of the patient population, ensuring effectiveness and inclusivity [50]. The IEDB population coverage tool (http://tools.iedb.org/population/) was utilized to evaluate the global distribution of *HIV-1* recognition of MHC class I and MHC class II alleles [50–52]. A list of CTL- and HTL-selected epitopes and identified alleles is given in Additional Tables 3 and 4.

### Construction of a multiepitope subunit vaccine

In the final vaccine construct, the qualified epitopes of each ORF and protein that were located in conserved domains and overlapped with other epitopes, unless there were limited numbers of epitopes, were considered. At the N-terminal end of the vaccine construct, the beta defensin-3 adjuvant (GIINTLQKYYCRVRGGRCAVLSCLPKEEQIGKCSTRGRKCCRRKK) was incorporated into the vaccine model to not only protect the construct from degradation [53] but also stimulate robust immune reactions, especially mucosal immune responses toward *HIV* and other viral infections [54]. This sequence is followed by the EAAAK linker and universal PADRE (AKFVAAWTLKAAA) to overcome the problems caused by highly polymorphic HLA class 2 alleles in the final vaccine construct [55]. GGGS linkers were used to join CTL epitopes, whereas GPGPG and KK linkers were used to join HTL and B-cell (BCL) epitopes, respectively. Finally, the C-terminal invasin sequence of *Yersinia* (TAKSKKFPSYTATYQF) was added to the C-terminus of the construct via the EGGE linker. Although the EAAAK linker can be characterized as a rigid linker in the final vaccine candidate, GGGS, GPGP, KK, and EGGE consequently provide flexibility for fusing the epitopes [56]. The abovementioned linkers were utilized to ensure the effective separation of individual epitopes [57]. Finally, the sequence of the vaccine model was screened through the VaxiJen server, AllerTOP v.2.0 server, ToxinPred2 webserver (https://webs.iiitd.edu.in/raghava/toxinpred2/batch.html), TMHMM server, and PIR peptide matching program [38, 39].

### Prediction of physicochemical and immunogenic properties

The physicochemical properties of the vaccine construct were characterized via ProtParam, a tool available on the ExPASy server [22, 58]. Using this tool, various properties, including molecular weight, theoretical pI, half-life in mammalian reticulocytes, yeast, and *Escherichia coli*, the instability index, the extinction coefficient, the amino acid composition, the atomic composition and GRAVY, were evaluated. Furthermore, the antigenicity and allergenicity of the multiepitope vaccine construct were investigated via VaxiJen and AllerTOP, respectively (Additional Table 14).

### Solubility of the antigenic fusion protein

The solubility of the chimeric vaccine was examined using the Protein-sol server (https://protein-sol.manchester.ac.uk/). The average solubility for the experimental dataset (PopAvrSol) is 0.45. Therefore, any scaled solubility value exceeding 0.45 is anticipated to indicate a higher solubility than the typical soluble *E. coli* protein from the experimental solubility dataset [59, 60].

### Secondary structure prediction, tertiary structure (3D) modeling, refinement, and validation of the vaccine construct

The sequence of the vaccine was submitted to the Self-Optimized Prediction Method with Alignment (SOPMA) server (https://npsa-prabi.ibcp.fr/NPSA/npsa_sopma.html) to predict four conformational states, including beta sheets, bridges, turns, and coil helices [61, 62]. Another tool used to evaluate the secondary structure of vaccine constructs was RNAfold (http://rna.tbi.univie.ac.at/cgi-bin/RNAWebSuite/RNAfold.cgi) [16, 63]. To develop the tertiary structures of the vaccine constructs, the I-TASSER (https://zhanggroup.org/I-TASSER/), Robetta (https://robetta.bakerlab.org/), and AlphaFold (https://usegalaxy.eu/?tool_id=alphafold) servers were used to generate five models [64, 65], and the optimal crude 3D models of the vaccine sequence were subsequently submitted to the GalaxyWEB server (https://galaxy.seoklab.org/) to rebuild unreliable termini or loops of the initial model structures to generate five refined models [66, 67]. The GalaxyWEB server provides five models that are determined based on various parameters, such as GDT-HA, RMSD, MolProbity, Clash score, Poor rotamers, and Rama favored. These models are used for template selection, sequence alignment, model building, and refinement. However, the server cannot determine the best model among the five proposed models. Therefore, the ERRAT (http://services.mbi.ucla.edu/ERRAT) [68], ProSA-Web (https://prosa.services.came.sbg.ac.at/prosa.php) [69], and RAMPAGE (http://mordred.bioc.cam.ac.uk/~rappe r/rampa ge.php) [70, 71] online tools were utilized to identify the optimal model in this research. The ERRAT program calculates the overall quality factor on the basis of the number of nonbonded interactions between different atomic types within a specific distance (0.35 nm); typically, models with a quality factor above 85 are considered good. The RAMPAGE tool uses a Ramachandran plot to assess the stereochemical quality of a protein structure. A better model will have more residues in the favored region and fewer residues in the disallowed region of the plot. The Z score from the ProSA-Web server is an indicator of the overall quality of the model, where a positive Z score suggests potential issues or errors in the structure [14, 39, 72].

### Disulfide engineering of the vaccine construct

Disulfide engineering is a biotechnological technique that involves mutation of a cysteine residue to create new disulfide bonds within a specific highly flexible protein region. Disulfide linkages exhibit remarkable stability and assist in the preservation of protein geometric conformations [73]. Disulfide engineering of the vaccine construct was carried out using disulfide via the Design 2 online tool (http://cptweb.cpt.wayne.edu/DbD2/). The server identifies the potential locations within the protein structure that have a greater likelihood of forming disulfide bonds [74].

### Prediction of discontinuous B-cell epitopes

The ElliPro tool (http://tools.iedb.org/ellipro/) of the IEDB server [75, 76] suggested the presence of linear and conformational B-cell epitopes in the constructed conjugated vaccine with a screening threshold of 0.5. The tertiary structure of the validated vaccine construct was submitted to the server, and epitopes with scores greater than the threshold were considered linear or discontinuous B-cell epitopes [58].

### Peptide‒protein molecular docking

The presentation of pathogen-derived peptides to T cells relies heavily on MHC molecules [39]. AutoDockVina software was used to assess the molecular docking process of the interaction performance of the anticipated epitopes (CTLs and HTLs) with their corresponding binding alleles [77]. To accomplish this, the protein database (PDB) website (https://www.rcsb.org/) was used to retrieve the PDB files of the most prevalent alleles (MHC molecules) (Additional Tables 3 and 4). To model the three-dimensional structure of T-cell epitopes as ligands, we utilized the PEPFOLD 4.0 server (https://mobyle2.rpbs.univ-paris-diderot.fr/cgi-bin/portal.py#forms::PEP-FOLD4) [78, 79].

### Protein‒protein docking between TLR3, TLR4, and TLR9 and the vaccine construct

Establishing a stable connection between a potential vaccine and an immune receptor is crucial for triggering effective immune responses. TLRs not only play crucial roles in the initial defense against pathogens but also serve as vital links between innate and adaptive immunity [39]. In other words, TLR3 recognizes double-stranded RNA (dsRNA) and single-stranded RNA (ssRNA) [80, 81]. However, TLRs can also bind to polyproteins and epitopes [82–84]; for example, TLR3 serves as a receptor for the construction of a vaccine that is capable of identifying the virus [29, 85–88]. For this purpose, the vaccine includes a TLR3 agonist called β-defensin, which is attached to the N-terminus via an EAAAK linker [88], and TLR4 is capable of triggering immune responses against viruses by recognizing proteins on the surface of the virus [39]. Molecular docking is a computational method that can be used to evaluate the binding affinity between vaccine constructs and immune receptors, as well as the formation of interaction complexes [79]. Consequently, we utilized the ClusPro 2.0 online server (https://cluspro.bu.edu/login.php) [89] to carry out molecular docking of the vaccine constructs with TLRs (TLR3, TLR4, and TLR9). The PDB files for TLR3 (PDB ID: 1ZIW), TLR4 (PDB ID: 3FXI), and TLR9 (PDB ID: 3 WPB) were extracted from the RCSB database (https://www.rcsb.org/) [14]. In this study, molecular docking was used for blind docking.

### Molecular dynamic simulation

Molecular dynamics (MD) simulation is widely acknowledged as a highly effective approach for the molecular examination of biological systems [90]. Here, the behavior of the designed vaccine against the TLR3, TLR4, and TLR9 systems was studied using Linux-based GROMACS software (http://www.mdtutorials.com/gmx/lysozyme/) [91]. During the initial phase of preparation, the vaccine-TLR complexes underwent the incorporation of OPLS-AA (Optimized Potential for Liquid Simulation) force field parameters. This step results in the generation of coordinate and topology files for the complex system. Afterward, the systems were solvated using the transferable intermolecular potential 3P (*TIP3P*) water model; then, the systems were neutralized with Cl- ions, ensuring that the topological and structural coordinates remained stable [39]. Subsequently, the process of energy minimization was conducted, resulting in the acquisition of the final structure through energy minimization (EM) [72]. The NVT ensemble equilibration lasted 100 ps and involved 50,000 steps to achieve the desired temperature. The NPT ensemble, with 50,000 steps, was then utilized to examine the density, potential, pressure, and temperature of the stabilized vaccine construct throughout the entire process. Following equilibration, MD simulations of 100 ns and 50,000,000 steps were conducted on the construct. The backbone’s root mean square deviation (RMSD) of energy was minimized after the MD simulation, and the results are presented in the form of graphs. Additionally, the radius of gyration, hydrogen bonds, and density plots were analyzed, as was the performance of the root mean square fluctuation (RMSF) protocol during the MD simulation.

### Immune stimulation

To conduct an immune simulation analysis of the *HIV-1*-specific vaccine, we employed C-IMMSIM (https://kraken.iac.rm.cnr.it/C-IMMSIM/index.php?page=1) [92]. This tool uses the Miyazawa-Jernigan residue-residue potential to evaluate the strength of the interaction between a T-cell receptor and a specific peptide-HLA complex [93]. C-IMMSIM was utilized with its default settings [72], except for host HLA selection; specifically, HLA-A*0101, HLA-A*0301, HLA-B*1501, HLA-B*0801, HLA-DRB1*1101, and HLA-DRB1*0101 were chosen because of their higher genotypic frequencies in the global population, as determined by the IEDB analysis server.

### Codon optimization and *in silico* cloning

**Fig. 1:**
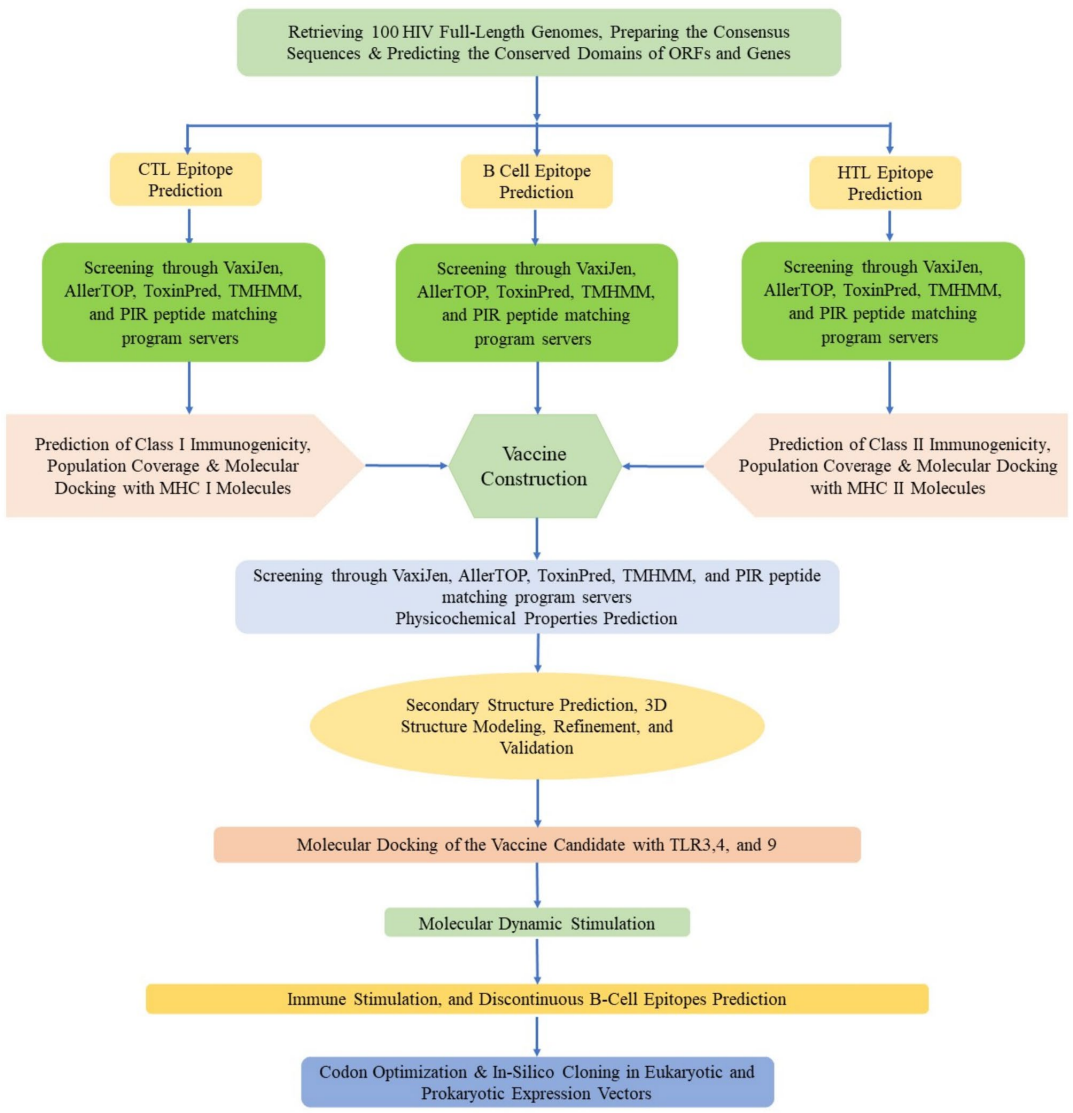
Overview of the flowchart of multiepitope vaccine design in this study

Since the epitopes of the vaccine model were compatible with both the human and mouse immune systems, both eukaryotic and prokaryotic expression vectors were generated. For constructing the adenovirus-based vaccine, the Kozak sequence containing a start codon that controls the translation initiation was incorporated at the N-terminus end of the vaccine protein. The level of MMLV-*RT* protein expression was enhanced by codon optimization performed by the VectorBuilder server (https://en.vectorbuilder.com/tool/codon-optimization.html) for the *Homo sapiens* expression system [94]. The cleavage sites of two restriction enzymes (*EcoRV* and *BglII*) were removed from the optimized cDNA sequence. A highly efficient reverse-transcribed nucleotide sequence with a suitable CAI and GC content was inserted into the pAdTrack-*CMV* shuttle vector between the *EcoRV* and *BglII* restriction sites under the control of the *CMV* promoter using SnapGene v7.1.1 [95]. In addition, similar processes were performed for *in silico* cloning of the prokaryotic vaccine construct in the pET28(a) plasmid using the *BamH1* and *Xho1* restriction enzymes and codon optimization in the K12 strain of *E. coli*. The flowchart of the vaccine design is illustrated in Fig. 1.

## Results

### Protein sequence retrieval and alignment

Up to June 2023, the frequency of subtypes and CRFs submitted to the LANL database and the accession numbers of 100 full-length *HIV* sequences of the most predominant subtypes and CRFs used in this study are presented in Additional Fig. 1 and Additional Table 5, respectively. The consensus sequences of *Gag*,* Env*,* Pro*,* IN*,* RT*,* Tat*,* Nef*,* Rev*,* Vif*,* Vpr*, and *Vpu* are displayed in Additional Table 6.

### Identification of conserved domains and the biophysical and biochemical features of HIV genes

The conserved domains of each sequence are listed in Additional Table 7. Among the different epitopes that passed through the various filters, only those located in the conserved domains were qualified for consideration in the vaccine construct. The physicochemical properties (i.e., the aliphatic index, grand average hydropathicity, instability index, molecular weight, and theoretical pI) of all the consensus sequences determined with the ExPASy ProtParam tool are shown in Additional Table 8.

### Linear BCL epitope prediction

Out of thousands of suggested epitopes, only a limited number with suitable features, such as antigenicity, topology, toxicity, and allergenicity, were selected (Additional Table 9). Finally, one epitope of each ORF and protein that overlapped with either other B-cell epitopes or CD8 and HTL epitopes was considered in the vaccine construct (Table 1). Additional Fig. 2 illustrates the predicted B-cell epitope of the Tat protein determined with the IEDB linear epitope tool.

**Table 1:** A list of B cell-selected epitopes of HIV-1 genes

Genes	Epitope	Software	Start	End	Score	VaxiJen Score	Allergenicity	Toxicity	Topology (With or without signal sequence)
**Gag**	IRLRPGG	Karplus	19	25	1.025	2.1646	Non- allergen	Non-toxin	Without
**Pro**	IKIGGQL	Karplus	13	19	1.046	1.0806	Non- allergen	Non-toxin	Without
**RT**	IPSINNE	Karplus	167	173	1.042	0.7476	Non- allergen	Non-toxin	Without
**IN**	SGYIEAE	Parker	81	87	2.857	0.7208	Non- allergen	Non-toxin	Without
**Vif**	SIEWRKR	Parker	86	92	1.486	3.4666	Non- allergen	Non-toxin	Without
**Vpr**	CQHSRIG	Parker	76	82	2.557	1.4496	Non- allergen	Non-toxin	Without
**Tat**	TKGLGIS	Parker	40	46	1.657	1.2613	Non- allergen	Non-toxin	Without
**Rev**	PAEPVPL	Karplus	67	73	1.024	0.9032	Non- allergen	Non-toxin	Without
**Vpu**	IDRIRER	Parker	43	49	1.029	0.6271	Non- allergen	Non-toxin	Without
**Env**	HYCAPAG	Parker	206	212	1.943	1.0720	Non- allergen	Non-toxin	Without
**Nef**	MRRTEPA	Karplus	20	26	1.05	1.5441	Non- allergen	Non-toxin	Without

### HTL epitope prediction

Epitopes that were proposed by both the IEDB MHC II and HLA binding webservers and were able to bind to at least one mouse H-2-I allele and one human allele were screened through a number of filters. The epitopes could bind to both human and mouse alleles simultaneously to induce immune responses in both mice and humans. Finally, only those epitopes that were antigenic, nonallergenic, nontoxic, and without signal sequences that can induce IFN-γ and Th1 humoral responses were considered in the final vaccine models (Table 2 and Additional Table 10); however, the *Rev* and *Vpr* proteins did not contain any subtitle epitopes, which might be due to the short length of these proteins.

**Table 2:** Potential antigenic and immunogenic HTL epitopes

Gene	Start	End	Epitope	Topology (With or Without signal sequence)	VaxiJen Score	Allergenicity	Toxicity	IFN-γ	Immunogenicity
**Gag**	10	24	GGKLDRWEKIRLRPG	Without	0.4991	Non-Allergen	Non-Toxin	+	0.3008
**Pro**	39	53	PGKWKPKMIGGIGGF	Without	1.0701	Non-Allergen	Non-Toxin	+	0.0796
**RT**	158	172	DFRKYTAFTIPSINN	Without	0.6093	Non-Allergen	Non-Toxin	+	0.0955
**IN**	25	39	DFNLPPVVAKEIVAS	Without	0.9722	Non-Allergen	Non-Toxin	+	0.1541
**Vif**	84	98	GVSIEWRKRRYSTQV	Without	1.5905	Non-Allergen	Non-Toxin	+	0.1403
**Tat**	43	57	LGISYGRKKRRQRRR	Without	1.2098	Non-Allergen	Non-Toxin	+	0.3977
**Vpu**	24	38	TIVFIEYRKILRQRK	Without	0.5875	Non-Allergen	Non-Toxin	+	0.2951
**Env**	204	218	PIHYCAPAGFAILKC	Without	0.4874	Non-Allergen	Non-Toxin	+	0.1947
**Nef**	11	25	VGWPAVRERMRRTEP	Without	0.9814	Non-Allergen	Non-Toxin	+	0.3567

### CTL epitope prediction

Among the vast majority of epitopes that interacted with 12 MHC supertypes, a small percentage were immunogenic, antigenic, nontoxic, nonallergenic, and nonhomologous to the human proteome and exhibited appropriate topology (Table 3 and Additional Table 11). Again, epitopes located in conserved domains were considered in the final vaccine construct (Additional Table 4).

**Table 3:** Potential antigenic and immunogenic CTL epitopes

Gene Name	Start	End	Epitope	Topology (With or Without signal sequence)	VaxiJen score	Allergenicity	Toxicity	Immunogenicity
**Gag**	31	39	LKHIVWASR	Without	1.7391	Non allergen	Non toxin	1.7391
**Pro**	12	20	TIKIGGQLK	Without	1.1453	Non allergen	Non toxin	1.1453
**RT**	171	179	NNETPGIRY	Without	0.4586	Non allergen	Non toxin	0.4586
**IN**	96	104	ETAYFILKL	Without	0.5370	Non allergen	Non toxin	0.5370
**Vif**	145	153	LQYLALTAL	Without	1.2523	Non allergen	Non toxin	1.2523
**Vpr**	52	60	DTWAGVEAI	Without	0.5053	Non allergen	Non toxin	0.5053
**Tat**	42	50	GLGISYGRK	Without	2.1524	Non allergen	Non toxin	2.1524
**Rev**	65	73	GRPAEPVPL	Without	0.9323	Non allergen	Non toxin	0.9323
**Vpu**	24	32	TIVFIEYRK	Without	1.7899	Non allergen	Non toxin	1.7899
**Env**	669	677	GLRIVFAVL	Without	0.8764	Non allergen	Non toxin	0.8764
**Nef**	188	196	RLAFRHMAR	Without	0.9852	Non allergen	Non toxin	0.9852

Despite the diversity of the HIV sequence, the conserved domains of ORFs and genes were highly similar among the consensus sequences obtained from the first, second, and third databases (in all subtypes and CRFs). Furthermore, our results revealed that the sequences of the predicted epitopes were highly similar to the sequences of all the subtypes and CRFs. Our research revealed that a substantial proportion of the CD4, CD8, and B-cell epitopes selected for the final vaccine contract were either 100% identical or had only a few mismatches with each other (Additional Table 12).

### Population coverage

**Fig. 2:**
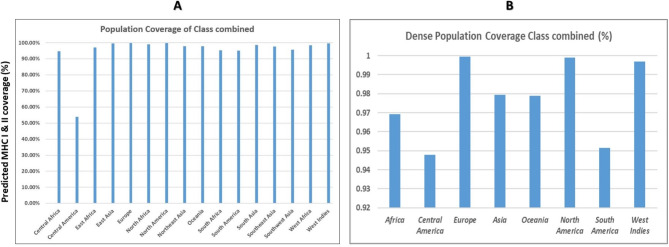
Percentage of the combined coverage of the selected CTL and HTL epitopes. **A**) Global population; **B**) dense population

The IEDB population coverage tool was utilized to analyze the population coverage of eleven CTL and nine HTL epitopes, along with their corresponding HLA alleles, across 16 and 8 different regions worldwide. European countries demonstrated the highest prevalence of the MHCI and II alleles, with 99.96% coverage for all the genes. Conversely, Central American countries had the lowest distribution (53.8%) of combined alleles across all the genes. Additionally, an examination of the effects of these epitopes on the population demonstrated that 95.04% of the global population (MHC class I and II combined) was covered. This information is visually represented in Fig. 2A-B and Additional Table 13. The findings on population coverage imply that the designed *HIV-1* vaccine candidates possess the ability to fight the global prevalence of *HIV* infection.

### Vaccine construction

**Fig. 3:**
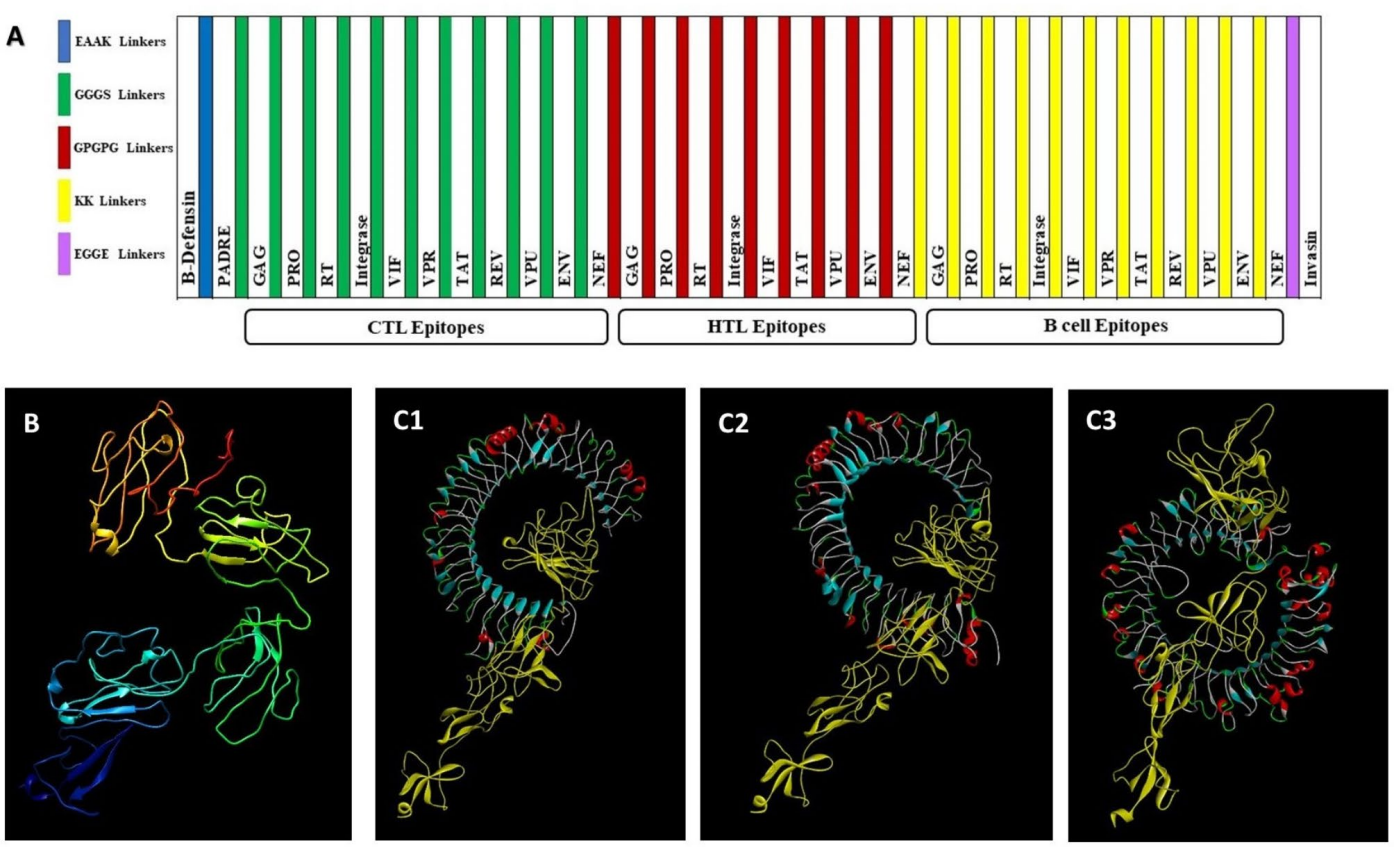
Graphical map, tertiary structure, and ligand‒receptor docked complex of the HIV-vaccine construct. **A**) The vaccine constructs consisted of an adjuvant, as well as CTL, HTL, and BCL epitopes, arranged from left to right; **B**) Model-1 was constructed with I-TASEER software (protein modeling service) after refinement with the Galaxy Web server; **C**) Docked complex of the vaccine construct with C1) TLR-3, C2) TLR-4, and C3) TLR-9. The vaccine molecule is indicated in yellow, while TLR-3, TLR-4, and TLR-9 are indicated in blue. The lowest energy scores of the vaccine construct with other TLRs were − 1284.4 for TLR3, -1381.5 for TLR4, and − 1355.9 for TLR, according to the ClusPro server

A multiepitope vaccine was constructed with eleven BCLs, eleven CTLs, and nine HTLs, which were joined together on the basis of the genomic arrangement of the HIV proteins. We failed to find qualified HLA epitopes for both the *Rev* and *Vpr* genes. To separate each CTL, HTL, and BCL epitope from the other epitopes, we added the GGGS, GPGPG, and KK linkers, respectively. These linkers ensure the efficient presentation of epitopes and maximal immunity in the body. Three adjuvants were incorporated into the vaccine construct. The first was beta defensin-3, a TLR4 agonist that was added at the N-terminal end of the construct. The second adjuvant was the PADRE sequence, which was fused to beta-defensin-3 via the EAAAK linker. To ensure maximal MHC-II allele coverage and CTL response induction, the PADRE sequence, which was separated from CTL epitopes by a GGGS linker, was considered in the construction. The third one was the C-terminal invasin sequence of *Yersinia*, and the BCL epitopes were linked to it by an EGGE linker (Fig. 3A).

On the basis of the diverse filters and servers, the sequence of the vaccine model (Additional Table 6) seems to be safe for in vitro and in vivo studies. The sequence of the vaccine model was antigenic, nontoxic, nonallergenic, and nonhomologous to the human proteome, without any signal sequence (Additional Table 14).

### Secondary and tertiary structure prediction, refinement, and validation

**Fig. 4:**
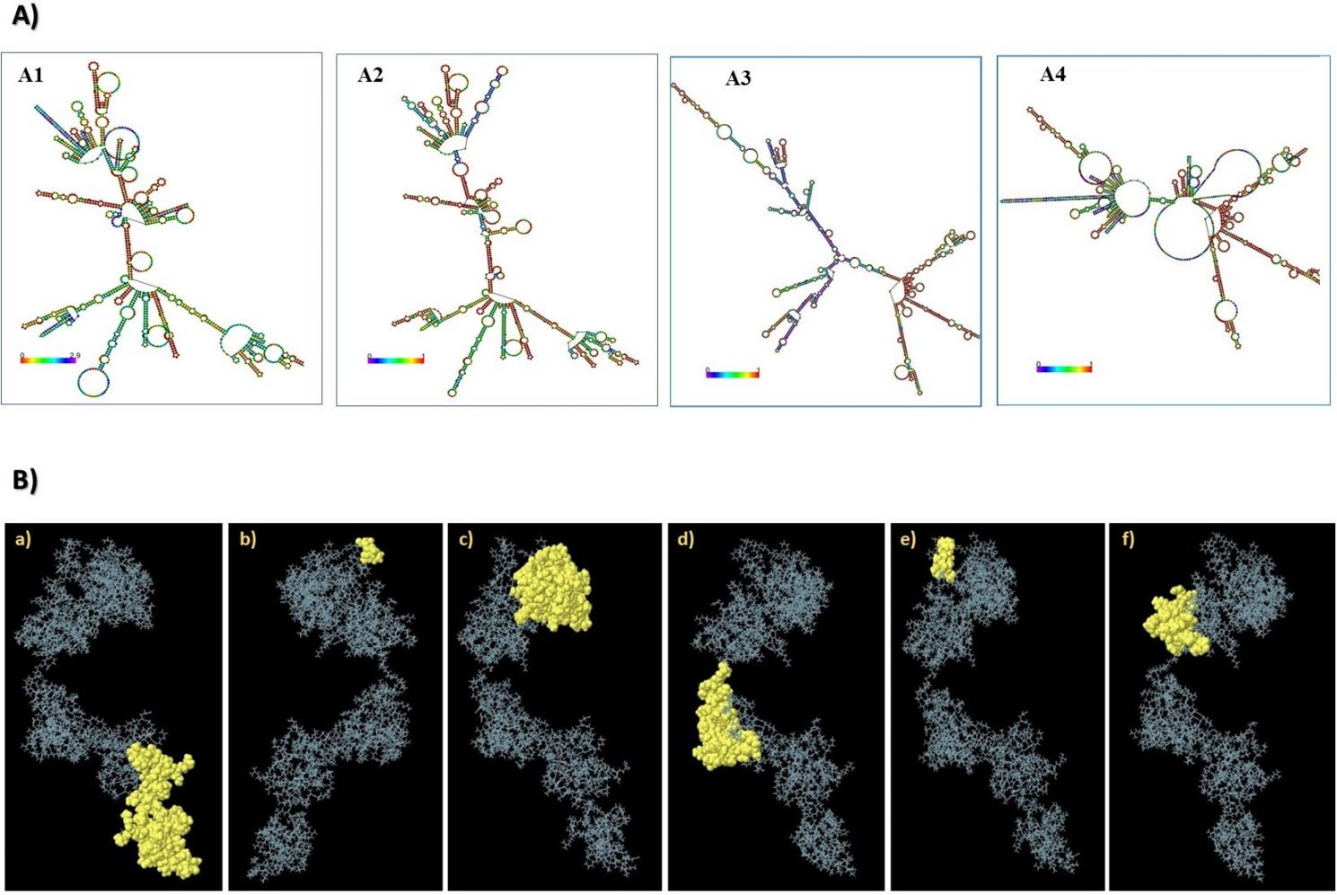
RNAfold results and the mapping of discontinuous B-cell epitopes on HIV vaccine constructs. **(A)** mRNA secondary structures in the human model (A1-2) and mouse model (A3-4). In general, the red color in the RNAfold software secondary structure figure usually denotes the highest probability of RNA base pairing, and the colors shifting from orange, yellow, green, blue, and violet to pink represent the probability of a reduction in base pairing within the secondary structure of the vaccine. **(B)** Mapping of discontinuous B-cell epitopes on the vaccine construct (a-f) is shown by the yellow region of the vaccine, which displays each discontinuous B-cell epitope containing residues 3 to 84 with score values ranging from 0.536 to 0.771

The SOPMA server was used to define the secondary structures of the ultimately chosen vaccine construct. The results (Additional Table 15) demonstrated that the proposed vaccine sequence included 14.12% alpha helices, 51.89% coils, and 8.75% beta sheets. Furthermore, the RNAfold website calculates the free energy of the structures of the optimized sequences of both the human and mouse vaccines, as illustrated in Fig. 4A1-4. The energies of the secondary centroid structures of the human and mouse vaccines were − 494.20 and − 384.60 kcal/mol, respectively, whereas the MFEs of the constructs during production were − 594.40 and − 585.70 kcal/mol, respectively. The kilocalorie per mole is a measurement unit for energy per atom, molecule, or similar particle. It is equivalent to one kilocalorie of energy per mole of a substance, defined as 1000 thermochemical gram calories [96]. To generate the 3D vaccine model, the I-TASSER, Robetta, and AlphaFold servers produced a total of 15 vaccine construct models, and the reliability of each model in I-TASSER was estimated by means of the C-score. Consequently, the model that demonstrated the most positive C-score (-1.50) was ultimately selected for refinement (Additional Fig. 3).

To refine the selected model, we utilized the GalaxyRefine server (Fig. 3B), and the quality analysis of the ProSA web server revealed an enhanced Ramachandran plot, where the greatest residues were found in the most favored area (Additional Fig. 4A). The results from the validation of the three servers were compared, and it was found that the I-TASSER model generated was more reliable than the other two methods (Additional Table 16). Overall, the three-dimensional high-quality model was selected as the 3D structure of the vaccine.

### Solubility of the vaccine

In our investigation, the solubility was 0.645, while its isoelectric point (PI) was 11.330. These findings demonstrated that the solubility of the fusion protein can be diminished through the inclusion of a linker between the antigenic compounds. The results are shown in Additional Fig. 4B.

### Disulfide engineering of the vaccine construct

During vaccine protein disulfide engineering, amino acid pairs were specifically chosen based on having a bond energy less than 2.2 kcal/mol. The vaccine design identified six pairs of amino acids meeting these criteria: 45 Lys and 48 Ala, 163 Tyr and 218 Glu, 179 Lys and 230 Gly, 220 Ile and 242 Ser, 231 Asp and 234 Lys, 266 Gly and 314 Tyr. The amino acid pairs chosen were used to create a mutant version of the original vaccine with disulfide bonds in the DbD2 online server (see Additional Table 17). The design of the *HIV* vaccine aimed to incorporate six potential pairs of amino acid residues that could form disulfide bonds. Hence, it can be regarded as a highly stable construct for a vaccine.

### Prediction of discontinuous B-bell epitopes

The presence of effective B-cell epitopes is vital in vaccine models for inducing humoral immunity against foreign pathogens [97]. ElliPro at the IEDB identified six epitopes with residues ranging from 3 to 79 in the vaccine construct and scores between 0.536 and 0.771 (Additional Table 18; Fig. 4B). The highest score was 0.735, with a residual position of 1–119 (Additional Fig. 5). In parallel, ten linear B-cell epitopes with residues ranging from 6 to 74 were confirmed in the vaccine, with scores ranging from 0.56 to 0.84 (Additional Table 19, Additional Fig. 6). Three linear epitopes of the vaccine construct overlapped with the discontinuous epitopes located in the vaccine model (Table 4).

**Table 4:** Discontinuous epitopes found in vaccine construct overlapped with linear epitopes

Protein	Overlapped Discontinuous Epitopes	Final Linear B cell epitopes	Start and end positions in vaccine construct	
**Pro**	A: S396, A: R397, A: I398, A: G399, A: K400, A: K401, A: T402	IKIGGQL	396–402	
**RT**	A: L405, A: G406, A: I407, A: S408, A: K409, A: K410, A: P411	IPSINNE	405–411	
**IN**	A: P414, A: V415, A: P416, A: L417, A: K418, A: K419, A: I420	SGYIEAE	414–420	

### Molecular docking of the T-cell epitopes with MHC molecules

In this investigation, we conducted a molecular docking process to examine the interactions between individual T-cell epitopes and their respective alleles, as outlined in Additional Tables 3 and 4. The results presented in Table 5 reveal that the binding energy between CTL epitopes and HLA I alleles varied from − 8.4 to -6.3 kcal/mol. In comparison, the binding energy range between the HTL epitopes and HLA II alleles was from − 7.3 to -5.1 kcal/mol, according to the AutoDock Vina docking data.

**Table 5:** Auto dock VINA results between CTL and HTL with the related alleles

		Gag	Pro	RT	IN	Vif	Vpr	Tat	Rev	Vpu	Env	Nef
**Binding Energy**	**CTL + HLA I**	-8	-6.3	-7.8	-6.8	-8.2	-7.9	-6.8	-6.6	-6.5	-8.4	-6.5
	**HTL + HLA II**	-5.9	-7.3	-6.5	-6.3	-5.1	NA	-5.5	NA	-6.4	-6.8	-6.2

NA: We did not find any suitable HTL epitopes for Vpr and Rev proteins

According to our data, the CTL epitope (GLRIVFAVL) of the envelope gene and the HTL epitope (PGKWKPKMIGGIGGF) of the protease gene had the highest binding energies when matched with the corresponding alleles (HLA-B*08:01 and HLA-DRB1*11:01).

### Protein‒protein docking between TLRs and vaccine constructs

The interaction between TLRs and the vaccine construct was examined through protein‒protein docking utilizing ClusPro 2.0. For each docking, a total of 30 models were generated (Additional Fig. 7). From this pool, we selected the models that effectively occupied the receptor with the most favorable energy scores. The lowest energy scores achieved for docking between the vaccine construct and TLR3, TLR4, or TLR9 were − 1284.4, -1381.5, and − 1355.9, respectively (Additional Table 20). The docked complexes revealed that the designed vaccine constructs exhibited the strongest binding affinity for the three TLRs at the lowest energy levels (Fig. 3C1-3).

### Molecular dynamic analysis

**Fig. 5:**
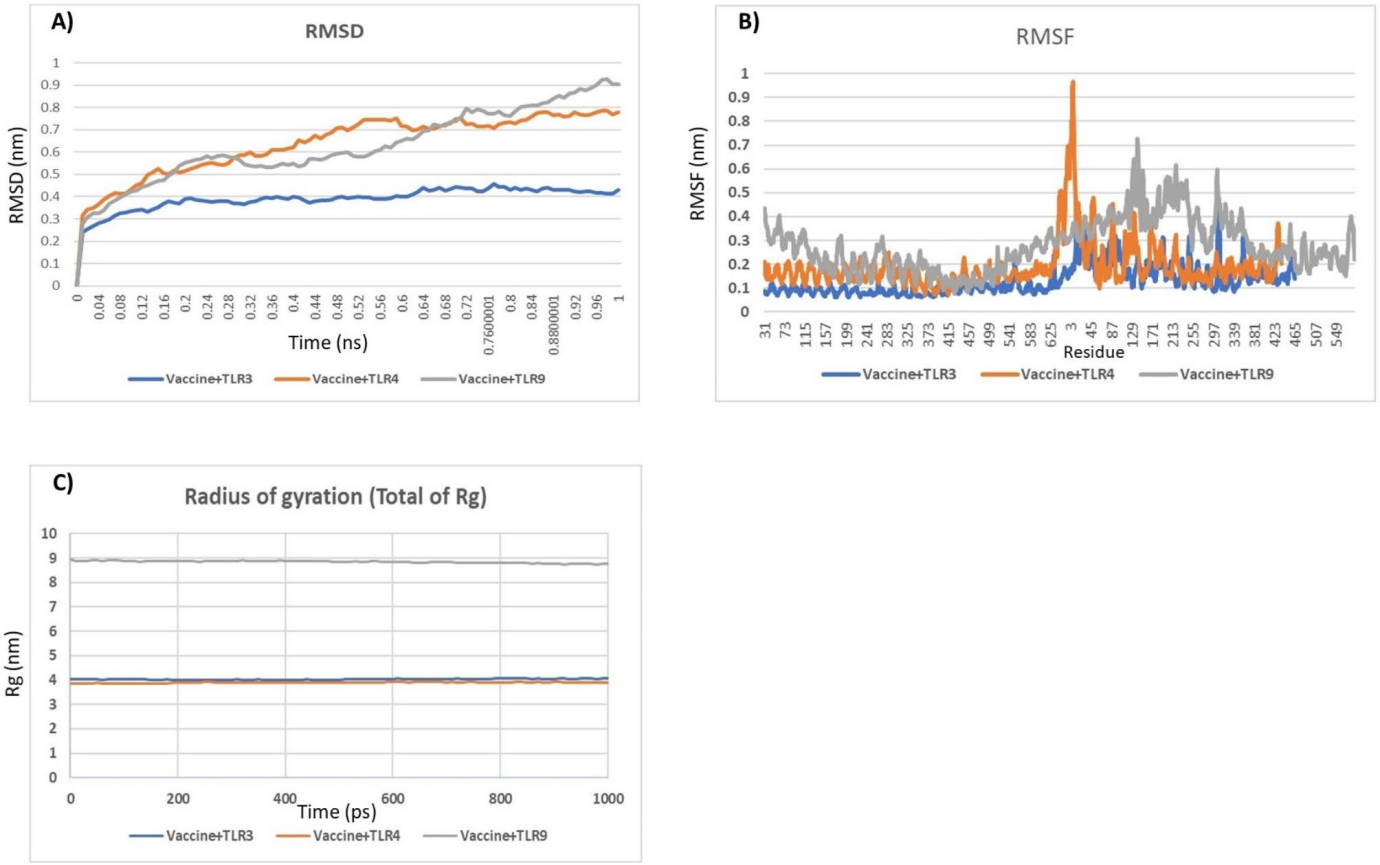
MD analysis of the TLR-3, TLR-4, and TLR-9 vaccine constructs. **A**) The RMSD of the docked complex exhibits a small deviation, reflecting the stable interaction between the vaccine construct and TLRs. **B**) The RMSF plot remained flat, reflecting the flexibility of the side chain of the docked protein complex. **C**) The radius of the gyration plot reveals a relatively flat curve, indicating the presence of a stable vaccine-TLR complex. In the plots, TLR3, TLR4, and TLR9 are indicated with blue, orange, and gray, respectively

Energy minimization, density, pressure, temperature, potential energy, and radius of gyration calculations were performed for the *HIV-1* vaccine construct. The radius of gyration provided evidence of the overall structural stability of the construct throughout the MD simulation (Fig. 5C). The RMSD and RMSF values were obtained by studying the trajectory generated after a 100 ns simulation. The designed vaccines with TLRs (TLR3, TLR4, and TLR9) exhibited RMSD values up to 0.78 nm, 0.98 nm, and 0.98 nm, respectively, indicating stability, with an average RMSD value of 0.73 nm for the vaccine during the simulation (Fig. 5B). The RMSF analysis confirmed that the overall structure of the protein remained stable throughout the MD simulation (Fig. 5A). This outcome suggested that the vaccine-TLR complex possesses considerable structural flexibility and stability, indicating a favorable response from the immune receptor.

### Immune stimulation

**Fig. 6:**
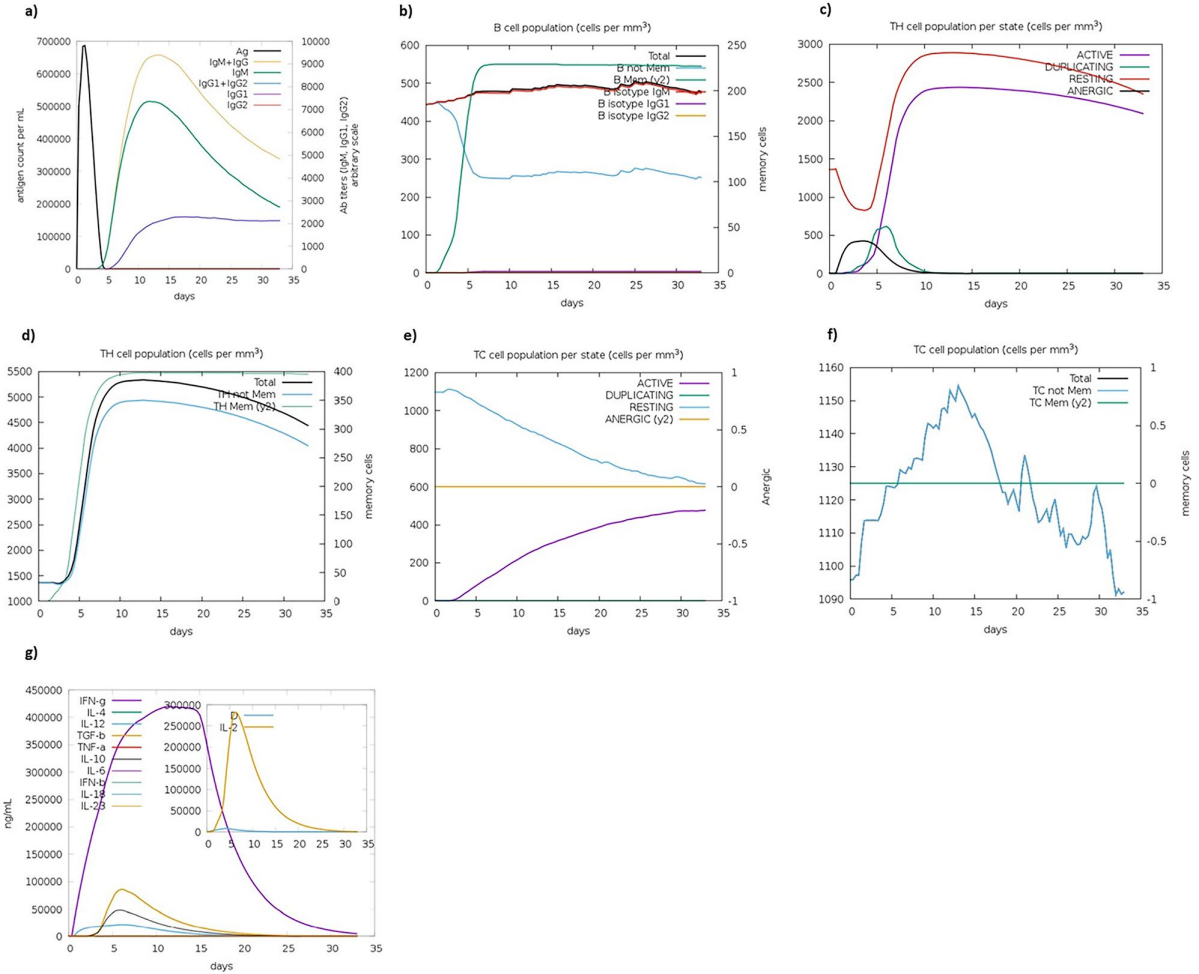
The immune simulation results obtained from C-IMMSIM after administering the vaccine construct were as follows: (**a**) The vaccine construct and immunocomplex trigger the production of various types of immunoglobulins. (**b**) Noticeable increase in the population of B cells, (**c**) significantly elevated population of active Th cells per state, (d) increased population of total Th cells, (**d**) increased population of active T cytotoxic lymphocytes per state, (**f**) elevated in the T cytotoxic (TC) cell population, and (**g**) elevated concentrations of cytokines and ILs. The inset plot shows the presence of a danger signal and the production of a substantial amount of the leukocyte growth factor IL-2

The immune simulation results for the *HIV-1* vaccine construct are shown in Fig. 6a-g. According to the *in silico* results, vaccine injection resulted in several immune responses, including the production of IgM and IgG, the levels of B-cell and Th-cell populations, the concentration of cytokines, and the generation of Ab titers, particularly IgM + IgG. Simultaneously, a significant quantity of the IgG1 antibody was also generated, while no detectable levels of IgG2 or IgG1 + IgG2 were detected (Fig. 6a). This decrease in antigen levels was caused by the overall increase in the counts of B cells and T cells (Fig. 6b). Moreover, there was a noteworthy increase in the population of T lymphocytes (Fig. 6c), total number of Th cells, and total number of memory Th cells (Fig. 6d), as did the total number of B cells. Furthermore, an increase in the population of active TCs per state was also observed (Fig. 6e). Moreover, the TC cell population per state (cells per mm3) was remarkably high, indicating heightened responsiveness to the *in silico*-developed multivalent vaccine candidate (Fig. 6f). On the other hand, the simulation plot of cytokines revealed significantly elevated levels of IFN-γ and a substantial increase in the production of IL-2, TGFβ, and IL-10. The production of IL-12 was relatively low (Fig. 6g), indicating the ability of the candidate vaccine to generate a conspicuous and suitable immune response in computational system. Hence, the final vaccine construct effectively triggered robust anti-*HIV-1* immunity, which ultimately resulted in subsequent clearance of the antigen from the system.

### In silico cloning of the vaccine candidates

**Fig. 7:**
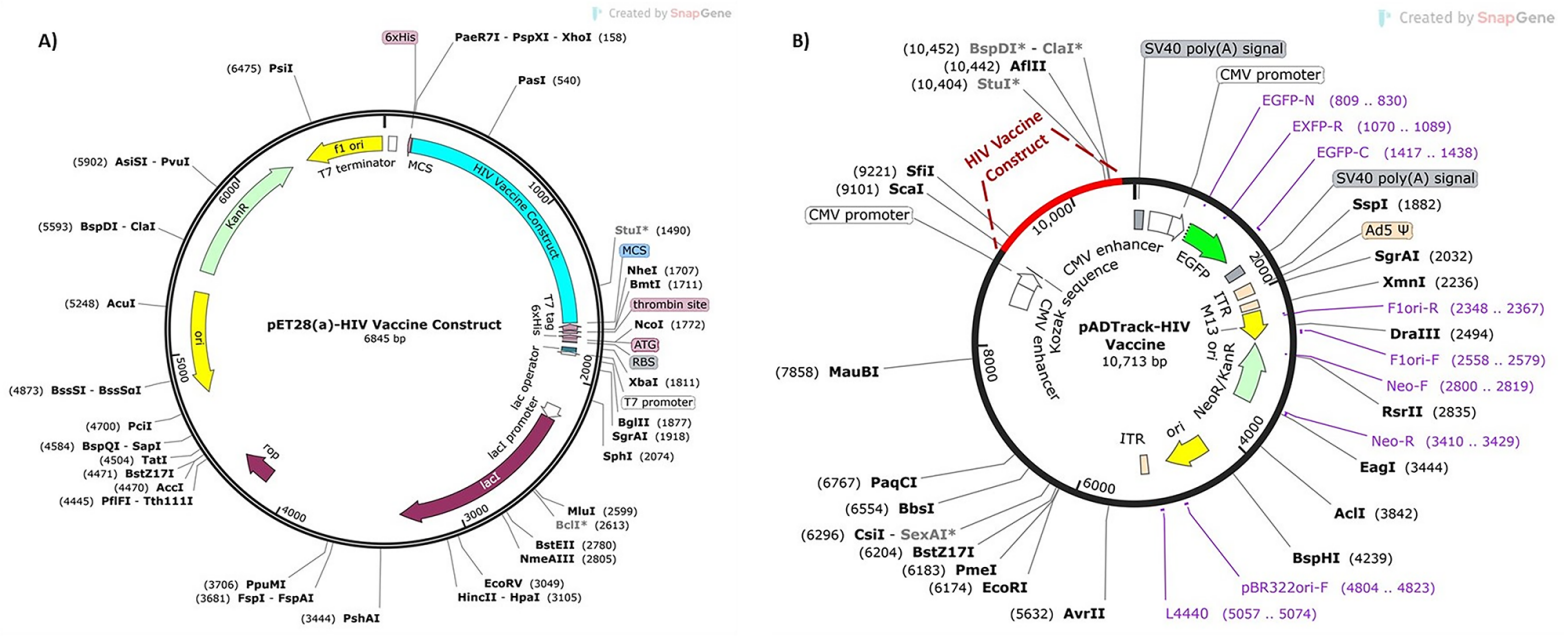
*In silico* cloning of the **A**) pET28 (a) plasmid and **B**) pAdTrack-*CMV* vector

The CAI and GC content of the optimized cDNA from humans and the K12 strain of *E. coli* were 0.91 and 59.86% and 0.93 and 59.86%, respectively, indicating the efficient expression of cDNA sequences in humans and *E. coli*. These optimized sequences (Additional Table 21) were inserted under the *CMV* promoter in the pAdTrack-*CMV* vector and pET28(a) plasmid through SnapGene software. To construct the pAdTrack-*CMV* vector containing the HIV vaccine sequence, the restriction site of *BglII* was inserted at the 5’ end of the vaccine sequence, which was subsequently inserted with the Kozak sequence. TAA, as the stop codon, was added at the 3’ end of the optimized codon, which was followed by the restriction site of *EcoRV* (Fig. 7B). To prepare pET28(a) containing the sequence of the *HIV* vaccine construct, we added the *Xho1* and *BamH1* restriction sites to the C and N termini of the optimized sequences of the vaccine model, respectively (Fig. 7A).

## Discussion

With the increasing occurrence of re-emerging and newly infectious diseases, medical researchers are continuously searching for effective and less expensive methods to treat infectious diseases. [98–102]. For example, vaccination is a method that protects people from contracting illness, leading to overall well-being and improved health [103, 104]. In recent years, multiepitope vaccines that can induce precise and robust immune responses have been proposed for many antigenic proteins [39, 105].

Invaluable research is revealing novel techniques to develop therapeutics against various human pathogens by utilizing bioinformatics methods. For instance, emerging pathogens such as henipavirus [106], *monkeypox* [107], and *SARS-CoV-2* [108], as well as drug-resistant zoonotic pathogens such as *Proteus penneri* (*P. penneri*) [109] and *Brucella melitensis* [110], for which specific vaccines are lacking or in need of enhancement, have been identified. This new paradigm of vaccine design promises shorter lead times, the preselection of peptide antivirals to prevent allergenic reactions, stability against mutational changes, and community-specific vaccine development.

In the case of *HIV*, multiepitope vaccines, particularly in the case of *HIV*, are limited by their ability to trigger an immune system against a few genotypes. For example, in a study conducted by Pandey et al., the designed vaccine elicited immunity only against *HIV* subtypes C and B [25].

The inefficacy of *HIV* vaccines is related to their inability to induce helper T-cell and cellular responses even in trials of the in silico vaccine EP *HIV*-1090 [13], their inability to produce vaccines against rapidly mutating *HIV* infection [25], their inability to produce broadly neutralizing antibodies, as suggested in three multiepitope vaccine trials in BALB/c mice [111], or their failure to activate the desired innate immune response and induce appropriate cytokines. To the best of our knowledge, only one invaluable report has described the design of an in silico vaccine against the whole *HIV* genome. In that report, the research group retrieved several *HIV* gene sequences from LANL regardless of prioritizing any *HIV* subtypes or CRFs. They performed several significant analyses and reported that both vaccine models were promising, but the vaccine construct with an adjuvant demonstrated better docking results and stability than those without an adjuvant [72]. Moreover, the epitopes were chosen from the conserved region of each protein or CRF. Upon investigating the full sequences of various HIV subtypes and CRFs, these identified epitopes were determined to be representative of the genetic diversity of HIV-1. As a result, the identified epitopes in the suggested vaccine model are highly conserved and can be representative across the HIV-1 genetic landscape, which can be effective for all HIV subtypes as well as CRFs to a significant extent.

The current *in silico* research designed distinctive vaccine models since the final epitopes are located in conserved domains of *HIV* genes that confer both cellular and humoral immune responses in mice and humans. Moreover, they targeted all the main *HIV* subtypes and CRFs, and the efficacy, stability, and toxicity of the vaccines were determined by different servers and tools.

*HIV-1* is known for its high genetic diversity; hence, the protein sequences of the strains of *HIV* can differ significantly. Therefore, the various *HIV* ORFs and proteins from the main subtypes and CRFs can improve the immunogenicity of the vaccine construct [112]. The *HIV* polyproteins and proteins that were analyzed in this report are essential for virus production and the detrimental effects of *HIV* infection in humans [113]; accordingly, such important proteins and polyproteins should be targeted in a vaccine model to completely suppress *HIV* infection [114]. Additionally, the final epitopes were selected from the conserved domains of all the proteins to improve the efficiency of the vaccine construct. The incorporation of highly conserved epitopes may elicit stronger and broader immune coverage against the main subtypes [115]. The epitopes included in the *in silico* study produce neutralizing antibodies against all *HIV* genes and induce appropriate IFN-ɣ and innate immune responses through interactions with TLR-3, TLR-4, and TLR-9.

B-cell epitopes play crucial roles in the development of an immune response that can resist viral infections. These epitopes have unique characteristics that enable B cells to identify and activate immune responses to a particular viral infection [116]. Linear BCL epitopes were produced and screened using multiple methods and filters, and this vaccine model has the potential to induce an effective immune response against HIV infection in computational systems.

One of the critical steps in triggering the immune response and developing memory cells against diseases is the presentation of antigens to CTLs through MHC-I/HLAI. The MHC-I/HLAI epitopes must possess substantial immunogenicity to activate CD8 + T lymphocytes. CTL cells are the main component of the MHC-I-mediated immune response and identify and kill damaged, virus-infected, or cancerous cells through the epitope presented by the MHC-I/HLAI molecule on the cell surface [117]. Since CTLs target various *HIV* proteins, all the proteins were screened to determine the immunogenic CD8 + T-cell epitopes. The HLA-I and HLA-II binding prediction tool generates a percentile rank as an affinity indicator for epitope-allele complexes. An indicator of percentile rank ≤ 1 (IC50 values < 50 nM) is considered a high-affinity peptide sequence, while intermediate-affinity peptide sequences show a percentile rank ≤ 10 (IC50 values < 500 nM) [118]. Significantly, peptides with a percentile rank of 10 or higher did not bind effectively [119]. Here, we set the base value of the percentile rank at 10 and 2 to evaluate epitope binding to all HLA class-I and class-II alleles. Additional Tables 3 and 4 show the selected epitopes incorporated in the vaccine construct that bind to both mouse and human alleles.

In this *in silico* report, potential HTL epitopes that induce IFN-ɣ were screened to trigger strong HTL immune responses through vaccination. A number of factors play a role in the outcome of infectious diseases and create a suitable response to a vaccine. These parameters can act at the virologic level, the physiology of the host, or at the molecular level [103, 120–125]. HTLs are also important key immune cells that acquire Th1 or Th2 phenotypes and stimulate immune responses, which activate macrophages, natural killer cells, and CD8 + T cells [29, 126, 127]. Furthermore, HTLs induce potent humoral and cellular responses by promoting the optimal expansion of CD8 + T cells and effective maintenance of CD8 + T cells [128]; hence, a clinical trial proposed that the CD4 + T-cell population is a potential vaccine candidate for eliciting robust immune responses against HIV infection [129]. Using advanced computational biology, the identification of the MHC-T cells that most strongly interact with the HLA complex is possible.

In the present study, distinctive filters were employed to screen all the primary epitopes. The ToxinPred server was used to check the toxicity of the peptide; from our data, the vaccine construct and final epitopes were safe and nontoxic. The other filter was VaxiJen v2.0, which indicates the ability of an antigen to induce an immune response to epitopes that are higher than the threshold of antigens in nature [31]. The antigenicity of the finalized epitopes and vaccine construct sequence were suitable for both in vivo and in vitro studies. The other factor that was assessed by filtration was immunogenicity, which refers to the ability of antigens to produce an immune response without binding to T cells. Therefore, both immunogenic and antigenic features are necessary for the efficacy of a vaccine [115]. Other characteristics of both the epitopes and the vaccine models that were estimated were nonallergenicity. Foreign antigenic substances trigger an immune response that leads to an allergenic reaction; therefore, safe human vaccines should be nonallergic [130]. Another quality that was screened was the presence of transmembrane helices and signal peptides in the epitopes according to the DeepTMHMM server. Those epitopes that consisted of either a transmembrane helix or signal peptide were excluded from the qualified epitope [131]. Interestingly, some of the epitopes chosen (PAEPVPL and IRLRPGG) in our research were found to be similar to those identified in previous studies, both in vivo and in vitro, which demonstrated effective inhibition of the virus [132, 133].

Population coverage analysis of the CTL and HTL epitopes demonstrated a coverage rate of 95.04% within the global population when considering both MHC Classes I and II (class combined). These findings suggest that our subunit vaccine, which consists of 20 epitopes, can provide coverage across a vast portion of the human population. According to our findings, the coverage in terms of geographical region was also exceptionally high, ranging from 94.79 to 99.96% for the vast majority of regions when the epitope set was considered, except for the Central American region, which presented a coverage rate of approximately 54%. The presence of outlier populations can be attributed to the limited knowledge regarding experimentally identified epitopes of HLA class I and II alleles, as well as the absence of detailed HLA typing in the allele frequencies of the populations included in the Allele Frequency Net Database. This discrepancy arises because the IEDB calculates population coverage based on HLA alleles that are typed to four digits, such as HLA A*01:01 [134].

Following the acquisition of all necessary criteria, 31 epitopes were joined together with different linkers. The application of the EAAAK linker in the vaccine construct guarantees the in vivo separation of epitopes in the natural environment [57, 135]. In addition, the adjuvant was joined to the multiepitope vaccine by the EAAAK linker to reduce the chance of interaction among functional domains [136]. A GGGS linker was used to separate CTL epitopes from individual CTL epitopes, as was the PADRE sequence. The KK linker joins BCL epitopes to conserve self-governing immunogenic responses [137]. Along with the advantages of a multiepitope subunit vaccine, appropriate adjuvants have been added to the construct to stimulate the immune response and overcome the low immunogenicity of such a vaccine [138–140]. It was revealed that defensin peptides play a crucial role as mucosal adjuvants in mouse models. Furthermore, a synthetic defensin adjuvant was added to the vaccine, resulting in increased cellular immunogenicity of the *HIV* peptide and a robust proliferative response [141]. The PADRE sequence is an adjuvant that was also used in a vaccine model to increase the stability of the construct and maximize the efficacy and potency of subunit vaccines that lead to better CTL responses [142, 143]. Moreover, the invasin peptide was used in combination with a vaccine construct to enhance the immune response efficiency of human adenovirus-based vaccines [144]. To recognize mRNA via ribosomes for stable expression of vaccine constructs in human cells, the Kozak sequence is recommended to be incorporated upstream of optimized cDNA [145]. Here, the Kozak sequence was also included in the vaccine model to improve the expression of the vaccine protein in human cells. According to the physicochemical parameters of the designed vaccine, these properties were desirable in the vaccine model.

The examination of the Ramachandran plot and various parameters for assessing the quality of the 3D model of the vaccine construct indicated that the refined 3D model of the final *HIV* vaccine construct was of favorable quality. Moreover, the results of the secondary and tertiary structure analyses revealed the high antigenic potential for the development of vaccines.

The Disulfide by Design 2.0 online tool predicted 53 pairs of amino acid residues with the ability to potentially create a disulfide bond. Indeed, disulfide bonds play a crucial role in stabilizing the geometric conformation of the sequence protein of a vaccine [146].

Molecular docking analysis demonstrated that the *HIV-1* vaccine construct had a strong affinity for TLRs (TLR3, TLR4, and TLR9), confirming that the human and mouse immune systems can effectively detect the multiepitope vaccine and trigger constant and strong immune responses in an *in silico* system. Previous research has indicated that TLRs play an essential role in the initiation of innate immune responses because they possess the ability to identify pathogens and trigger the development of the adaptive immune system [147]. For instance, TLR4 recognizes viral structural proteins and stimulates the production of inflammatory cytokines, whereas TLR3 initiates the activation of dendritic cells mediated by *HIV-1* [79]. According to previous reports, TLRs (2, 3, 4, 5, 8, and 9) can be activated by *HIV*, initiating downstream pathways that trigger the production of proinflammatory cytokines to combat *HIV* infection [14].

Codon optimization was performed in human and *E. coli* K12 hosts to improve transcriptional and translational efficiency. We selected the adenovirus and pET28a vectors to express the final vaccine protein in both prokaryotic and eukaryotic systems. Due to the degeneracy of the codons, each amino acid may match up to codons, and the choices of synonymous codon pairs are not the same in various species. In other words, different organisms and species prefer to use one or several specific synonymous codons called optimal codons [148].

MD simulation analysis confirmed the stability of the vaccine-TLR complex under diverse environmental conditions, including alterations in pressure and temperature. The preliminary assessment of the trajectory, including the computation of the RMSD, RMSF, radius of gyration, and hydrogen bonds, all concurred with the exceptional stability exhibited by the vaccine-TLR complexes within a biological environment. Additionally, compared to vaccine-receptor complexes, the TLR4 vaccine construct appears to be more stable when subjected to natural conditions.

Finally, the outcomes of the immune simulation acquired from the CIMMSIM interface revealed a decrease in the persistence of the elevated antigen after five days owing to the exponential production of IgM + IgG and IgM, which diminished over approximately 35 days. Moreover, the vaccine was found to increase the populations of Th cells, memory cells, and IL-2 + cells. Furthermore, we observed an increase in the number of dendritic cells, macrophages, and NK cells during immune stimulation with the designed vaccine. The immune simulation results confirmed that the vaccine effectively stimulated both innate and adaptive immune responses in our computational system.

Similar to other reports [29, 45], the main focus of this report was to develop a multivalent epitope-based vaccine to generate memory cells and induce chemokines upon exposure in both humans and mice, which was verified using a molecular simulation method. Consequently, epitopes with the ability to bind to both mouse and human alleles were chosen to induce immunogenicity in both species. Epitope selection from human epitopes does not necessarily result in stimulation of the immune system in mice, and vice versa. The recommended vaccine was tested for thermostability, stability, hydrophilicity, antigenicity, and non-allergenicity by immunoinformatic methods. In conclusion, the proposed vaccine models may be promising vaccine candidates for controlling *HIV* infection. In addition, 100 genomes are not representative of worldwide infections and thus HIV diversity; moreover, many recombinant forms are lacking, and drawing significant conclusions about HTL and CD8 + epitopes may not be applicable to all circulating subtypes and CRFs. Therefore, the primary steps of the methods included retrieving the HIV full genome, generating the consensus sequences of ORFs and genes and finding the conserved domains, which were subsequently repeated for 2 other sets of 100 HIV whole genomes obtained from two other datasets, NCBI and ENA. The three sets of HIV whole genomes were not redundant across the dataset but had similar subtypes to determine whether the epitopes predicted from the HIV genomes obtained from the LANA were confirmed. There were some variations in the consensus sequences; however, the conserved domains were remarkably similar across the consensus sequences obtained from different databases. The study revealed that a majority of the CD4, CD8, and B-cell epitopes identified for the vaccine contract displayed either complete similarity or minor discrepancies with each other. The challenge in eliminating HIV may not be linked to the variety in the HIV genome but rather to the integration of HIV into the host genome, which results in the persistence of the virus, the clonal expansion of infected cells, and the presence of long-lasting viral reservoirs that hinder eradication efforts.

The limitations of designing vaccines using bioinformatic tools are associated with the accuracy, suitability, and robustness of the bioinformatics software, tools, and online web servers used for each analysis and prediction. The suggested vaccine models need to be experimentally verified in the laboratory to ensure their safety and immunogenicity. Moreover, mRNA vaccines utilize modern technology, and the potential long-term side effects remain uncertain.

Since the onset of the SARS-CoV-2 pandemic, there has been a significant emphasis on utilizing mRNA as a vehicle for providing instructions for the synthesis of target proteins within host cells. A notable benefit of this approach is the ability to easily modify mRNA vaccines to target different diseases by altering the specific coding sequence for various proteins. Consequently, this adaptability makes mRNA vaccines particularly promising for advancing research on HIV vaccines. Future studies should consider the following levels of research: increasing the number of full-length HIV genomes with a balanced representation of subtypes and CRFs, assessing the effectiveness of the vaccine constructs on various strains of the HIV virus, enhancing the development of the proposed vaccine constructs in laboratory settings, and investigating the efficacy of the vaccine through various phases of clinical trials.

## Conclusions

The AIDS epidemic caused by *HIV* has been a global public health problem for more than 40 years, resulting in approximately 40 million deaths. There are two strategies to control this epidemic: treatment, for which many advances have been made, and vaccination, for which there is still no effective vaccine. Therefore, for the first time, vaccine models that may target major *HIV* subtypes and CRFs were designed using various novel immunoinformatic methods to help control *HIV* infection. The proposed vaccine constructs contained B and T-cell epitopes, which were characterized by high antigenicity, immunogenicity, nonallergenicity, nontoxicity, conservancy, nonhomology with the human proteome, and good binding affinity with their corresponding HLA alleles and TLR3, 4, and 9, with excellent population coverage. All the selected epitopes were located within conserved domains of two ORFs (*Gag* and *Env*) and nine *HIV* proteins (*Pro*, *IN*, *RT*,* Tat*,* Nef*,* Rev*,* Vif*,* Vpr*, and *Vpu*). The designed vaccine contained qualified epitopes and suitable adjuvants that can activate both humoral and cell-mediated immunity against *HIV-1*, which was confirmed by the immune stimulation method. The high expression rate of the vaccine models in human cells and *E. coli* bacteria was confirmed by performing codon optimization. The vaccine constructs showed good binding affinity for the immune receptors TLR3, TLR4, and TLR9. Finally, vaccine models are proposed as potential targets for in vitro and in vivo investigations of *HIV* infection.

## Electronic supplementary material

Below is the link to the electronic supplementary material.

Supplementary Material 1

Supplementary Material 2

Supplementary Material 3

Supplementary Material 4

Supplementary Material 5

Supplementary Material 6

Supplementary Material 7

Supplementary Material 8

## Data Availability

This study’s data generated and analyzed has been incorporated into this submitted article.
