# Correction: Reverse vaccinology approaches to design a potent multiepitope vaccine against the *HIV* whole genome: immunoinformatic, bioinformatics, and molecular dynamics approaches

**DOI:** 10.1186/s12879-024-09951-4

**Published:** 2024-09-20

**Authors:** Ava Hashempour, Nastaran Khodadad, Shokufeh Akbarinia, Farzane Ghasabi, Younes Ghasemi, Mohamad Matin Karbalaei Ali Nazar, Shahab Falahi

**Affiliations:** 1 HIV/AIDS Research Center, Institute of Health, Shiraz University of Medical Sciences, Shiraz, Iran; 2 Pharmaceutical Sciences Research Center, Shiraz University of Medical Sciences, Shiraz, Iran; 3 Department of Biotechnology, School of Pharmacy, Shiraz University of Medical Sciences, Shiraz, Iran; 4 Zoonotic Diseases Research Center, Ilam University of Medical Sciences, Ilam, Iran

Following publication of the original article [1], we have been notified that the authors missed adding the code of ethics to the paper: IR.SUMS.MED.REC.1403.371.
