# Supplementary material for: Reverse vaccinology approaches to design a potent multiepitope vaccine against the HIV whole genome: immunoinformatic, bioinformatics, and molecular dynamics approaches
Source: BMC Infect Dis. 2024 Aug 28;24:873. doi: 10.1186/s12879-024-09775-2 (PMC11360854; doi:10.1186/s12879-024-09775-2)
Supplement: Supplementary file 1 — Supplementary Material 1 [file 12879_2024_9775_MOESM1_ESM.docx]

**Supplemental Table 1.** The accession numbers of 100 full-length HIV genomes retrieved from NCBI

| **Accession Number** | **Subtype** | **Accession Number** | **Subtype** | **Accession Number** | **Subtype** |
| --- | --- | --- | --- | --- | --- |
| OQ121858.1 | G | OM209610.1 | B | MG760374.1 | AE |
| KX389648.1 | G | OM209715.1 | B | OQ357234.1 | AE |
| KX228812.1 | G | OM209815.1 | B | OQ121843.1 | AG |
| JX245014.1 | O | OM209915.1 | B | MT877448.1 | AG |
| MH705149.1 | O | OM209938.1 | B | MT877447.1 | AG |
| AB703613.1 | 35-AD | OM209982.1 | B | KC473840.1 | 47-BF1 |
| OK649294.1 | C | MW881699.1 | B | MN090740.1 | B |
| OM825587.2 | C | MW881697.1 | B | MT222951.1 | B |
| OM825657.2 | C | JF320227.1 | B | MT417770.1 | B |
| OM825583.2 | C | JN692479.1 | B | ON421479.1 | B |
| AB254151.1 | C | JN692461.1 | B | ON421467.1 | B |
| AF443090.1 | C | JN692465.1 | B | ON421471.1 | B |
| AY463218.1 | C | JF320245.1 | B | MW881670.1 | B |
| AY253304.1 | C | JF320225.1 | B | MW881665.1 | B |
| OK649292.1 | C | KF561441.1 | B | MT417752.1 | B |
| OM825579.2 | C | MT559050.1 | B | ON421464.1 | B |
| AY727522.1 | C | MT559061.1 | B | ON421468.1 | B |
| AY772692.1 | C | JQ341411.1 | B | ON421462.1 | B |
| AY878072.1 | C | MT559051.1 | B | MW881726.1 | B |
| DQ369983.1 | C | KR914678.1 | B | MN090737.1 | B |
| OM825580.2 | C | MZ642265.1 | A | MW262779.1 | B |
| MG365768.1 | F | MZ642264.1 | A | MW262771.1 | B |
| OM825095.2 | D | JX236677.1 | A | ON816966.1 | B |
| OM825096.2 | D | MZ642267.1 | A | ON816972.1 | B |
| OM825105.2 | D | KF716472.1 | A | OM209985.1 | B |
| MZ427717.1 | A6 | MT635147 | AE | MW881701.1 | B |
| MZ427734.1 | A6 | MT624756.1 | AE | MW881705.1 | B |
| OQ513525.1 | BC | AB253683.1 | AE | MW881708.1 | B |
| KF835545.1 | BC | ON902299.1 | AE | MW881709.1 | B |
| OP994768.1 | B | MH327751.1 | AE | OM209731.1 | B |
| OP994824.1 | B | ON816971.1 | B | OM209652.1 | B |
| OP994822.1 | B | MW262778.1 | B | OM209405.1 | B |
| OP994821.1 | B | OL872744.1 | B | OQ948507.1 | B |
| OP994815.1 | B |  |  |  |  |

**Supplemental Table 2.** The accession numbers of 100 full-length HIV genomes retrieved from ENA

| **Accession Number** | **Subtype** | **Accession Number** | **Subtype** | **Accession Number** | **Subtype** |
| --- | --- | --- | --- | --- | --- |
| OQ121873.1 | G | OM209611.1 | B | MG760375.1 | AE |
| KX389645.1 | G | OM209716.1 | B | ON959743.1 | AE |
| AF423760.1 | G | OM209818.1 | B | OQ121846.1 | AG |
| KF859742.1 | O | OM209919.1 | B | AB049811.1 | AG |
| MH705145.1 | O | OM209939.1 | B | AB052867.1 | AG |
| AB703611.1 | 35-AD | OM209983.1 | B | KC473839.1 | 47-BF1 |
| OK649291.1 | C | MW881692.1 | B | MN090739.1 | B |
| OM825589.1 | C | MW881713.1 | B | MN090738.1 | B |
| OM825660.1 | C | JF320224.1 | B | MT417767.1 | B |
| OM825590.2 | C | JN692478.1 | B | ON421480.1 | B |
| AB254152.1 | C | JN692462.1 | B | ON421465.1 | B |
| AF443092.1 | C | JN692468.1 | B | ON421473.1 | B |
| AY463219.1 | C | JF320243.1 | B | MW881660.1 | B |
| AY253303.1 | C | MH078552.1 | B | MW881659.1 | B |
| OK649290.1 | C | AY839827.1 | B | MT417754.1 | B |
| OM825576.1 | C | MT559053.1 | B | MT559044.1 | B |
| AY713416.1 | C | MT559062.1 | B | ON421498.1 | B |
| AY772693.1 | C | DQ054367.1 | B | ON421509.1 | B |
| AY878068.1 | C | MT559054.1 | B | MW881745.1 | B |
| DQ369984.1 | C | KR914675.1 | B | MW881746.1 | B |
| OM825581.2 | C | MZ642261.1 | A | MW405332.1 | B |
| MG365762.1 | F | JX236678.1 | A | MW262767.1 | B |
| OM825110.1 | D | AB253421.1 | A | ON816978.1 | B |
| OM825097.1 | D | AB253422.1 | A | ON816973.1 | B |
| OM825106.1 | D | AB287378.1 | A | OM209986.1 | B |
| MZ427720.1 | A6 | MT635149.1 | AE | MW881717.1 | B |
| MZ427731.1 | A6 | MT624757.1 | AE | MW881711.1 | B |
| OQ513522.1 | BC | AB253684.1 | AE | MW881716.1 | B |
| KF835544.1 | BC | ON902300.1 | AE | MW881718.1 | B |
| OQ948508.1 | B | MH327756.1 | AE | OM209732.1 | B |
| OQ948509.1 | B | ON816975.1 | B | OM209653.1 | B |
| OQ948511.1 | B | MW262780.1 | B | OM209655.1 | B |
| OQ948512.1 | B | OL872751.1 | B | OQ948514.1 | B |
| OQ948513.1 | B |  |  |  |  |

**Supplemental Table 3.** A list of HTL-selected epitopes and identified MHC alleles in HIV-1 genes

| **Gene** | **Mouse Allele** | **Epitope** | **Start** | **End** | **Antigenic**  **Score** | **MHC-II Allele (IEDB Percentile Rank ≤ 10)** | **PDB-ID** |
| --- | --- | --- | --- | --- | --- | --- | --- |
| **Gag** | H2-IEd | GGKLDRWEKIRLRPG | 10 | 24 | 0.4872 | HLA-DRB1*13:02, **HLA-DRB1*15:01**, HLA-DRB1*12:01, | **1BX2*** |
| **Pro** | H2-IEd | PGKWKPKMIGGIGGF | 39 | 53 | 0.3717 | HLA-DRB1*08:02, HLA-DQA1*04:01/DQB1*04:02, HLA-DPA1*01:03/ DPB1*02:01, **HLA-DRB1*11:01** | **6CPL** |
| **RT** | H2-IAb | DFRKYTAFTIPSINN | 158 | 172 | 0.2042 | HLA-DQA1*04:01/DQB1*04:02, HLA-DPA1*02:01/ DPB1*14:01, HLA-DRB1*07:01, HLA-DRB1*09:01, HLA-DQA1*01:01/ DQB1*05:01, HLA-DQA1*03:01/ DQB1*03:02, HLA-DRB1*04:05, **HLA-DRB1*15:01**, HLA-DPA1*03:01/DPB1*04:02, HLA-DRB1*04:01, HLA-DPA1*02:01/ DPB1*05:01 | **1BX2** |
| **IN** | H2-IAd | DFNLPPVVAKEIVAS | 25 | 39 | 0.0270 | HLA-DPA1*01:03/DPB1*02:01, HLA-DRB1*09:01, HLA-DRB1*08:02, **HLA-DRB1*01:01** | **1AQD** |
| **Vif** | H2-IEd | GVSIEWRKRRYSTQV | 84 | 98 | 0.4503 | HLA-DRB1*13:02, **HLA-DRB1*11:01** | **6CPL** |
| **Tat** | H2-IEd | LGISYGRKKRRQRRR | 43 | 57 | 0.5439 | HLA-DRB1*13:02, HLA-DRB1*03:01, **HLA-DRB1*11:01** | **6CPL** |
| **Vpu** | H2-IEd | TIVFIEYRKILRQRK | 24 | 38 | 0.8035 | HLA-DRB1*13:02, HLA-DRB1*08:02, HLA-DPA1*02:01/DPB1*05:01, HLA-DPA1*01:03/ DPB1*02:01, HLA-DRB1*11:01, HLA-DPA1*01:03/ **DPB1*04:01**, HLA-DQA1*04:01/DQB1*04:02, HLA-DPA1*03:01/ DPB1*04:02, HLA-DRB1*03:01, HLA-DRB4*01:01 | **5NI9** |
| **Env** | H2-IAb | PIHYCAPAGFAILKC | 204 | 218 | 0.0099 | **HLA-DRB1*01:01** | **1AQD** |
| **Nef** | H2-IEd | VGWPAVRERMRRTEP | 11 | 25 | 0.0521 | HLA-DQA1*01:01/DQB1*05:01, **HLA-DRB1*15:01** | **1BX2** |
| *** The bold font identified the Human HLA and the corresponding ID used in docking between epitopes and alleles.** | | | | | | | |

**Supplemental Table 4.** A list of CTL-selected epitopes and identified MHC alleles in HIV-1 genes

| **Gene** | **Mouse Allele** | **Epitope** | **Start** | **End** | **Antigenic**  **Score** | **MHC-I Allele (IEDB Percentile Rank ≤ 10)** | **PDB-ID** |
| --- | --- | --- | --- | --- | --- | --- | --- |
| **Gag** | H-2-Kd | LKHIVWASR | 31 | 39 | 0.0402 | HLA-A*33:01, HLA-A*31:01, **HLA-A*30:01**, HLA-A*68:01 | **6J1W*** |
| **Pro** | H-2-Qa1 | TIKIGGQLK | 12 | 20 | 0.4553 | **HLA-A*30:01**, HLA-A*03:01, HLA-A*11:01, HLA-A*68:01, HLA-A*31:01, HLA-A*33:01, HLA-A*30:02, HLA-A*26:01, HLA-A*32:01 | **6J1W** |
| **RT** | H-2-Kq | NNETPGIRY | 171 | 179 | 0.4260 | **HLA-A*01:01**, HLA-A*30:02, HLA-A*26:01, HLA-B*44:03, HLA-B*44:02, HLA-B*35:01, HLA-B*53:01, HLA-B*15:01, HLA-B*40:01, HLA-A*11:01, HLA-A*68:01 | **6MPP** |
| **IN** | H-2-Kb | ETAYFILKL | 96 | 104 | 0.9573 | HLA-A*68:02, HLA-A*26:01, HLA-A*32:01, **HLA-A*02:06**, HLA-B*51:01, HLA-A*68:01, HLA-B*53:01, HLA-B*58:01, HLA-A*01:01, HLA-B*57:01, HLA-A*33:01, HLA-B*08:01, HLA-B*35:01, HLA-A*02:03, HLA-A*02:01, HLA-A*23:01, HLA-A*24:02, HLA-B*44:03, HLA-B*44:02, HLA-A*11:01, HLA-A*30:02, HLA-B*40:01, HLA-B*07:02, HLA-A*31:01, HLA-A*30:01, HLA-A*03:01 | **3OXR** |
| **Vif** | H-2-Kb | LQYLALTAL | 145 | 153 | 0.6023 | **HLA-B*15:01**, HLA-A*02:06, HLA-B*08:01, HLA-B*40:01, HLA-A*02:03, HLA-A*32:01, HLA-A*02:01, HLA-B*07:02, HLA-B*35:01, HLA-B*51:01, HLA-A*30:02, HLA-A*23:01, HLA-A*26:01, HLA-A*24:02, HLA-A*30:01, HLA-B*44:03, HLA-B*44:02, HLA-B*53:01, HLA-A*68:02 | **6UZP** |
| **Vpr** | H-2-Kq | DTWAGVEAI | 52 | 60 | 0.7026 | HLA-A*68:02, **HLA-B*51:01**, HLA-A*26:01, HLA-B*53:01, HLA-B*35:01, HLA-A*02:06, HLA-A*32:01, HLA-A*33:01, HLA-A*02:01, HLA-A*68:01, HLA-B*58:01, HLA-B*57:01, HLA-A*02:03, HLA-B*08:01, HLA-B*40:01, HLA-A*01:01, HLA-A*24:02 | **1E28** |
| **Tat** | H-2-Qa1 | GLGISYGRK | 42 | 50 | 0.2220 | **HLA-A*03:01**, HLA-A*11:01, HLA-A*30:01 | **7L1C** |
| **Rev** | H-2-Dd | GRPAEPVPL | 65 | 73 | 0.0075 | **HLA-B*40:01**, HLA-A*24:02, HLA-A*23:01, HLA-B*08:01, HLA-B*07:02 | **6IEX** |
| **Vpu** | H-2-Kb | TIVFIEYRK | 24 | 32 | 0.8185 | **HLA-A*68:01**, HLA-A*11:01, HLA-A*03:01, HLA-A*33:01, HLA-A*31:01, HLA-A*30:01, HLA-A*26:01, HLA-A*32:01, HLA-A*68:02 | **6PBH** |
| **Env** | H-2-Kb | GLRIVFAVL | 669 | 677 | 0.1227 | **HLA-B*08:01**, HLA-A*02:03, HLA-B*07:02, HLA-B*15:01, HLA-A*32:01, HLA-A*02:01, HLA-A*30:01, HLA-A*02:06 | **7NUI** |
| **Nef** | H-2-Qa1 | RLAFRHMAR | 188 | 196 | 0.8188 | HLA-A*31:01, **HLA-A*03:01**, HLA-A*33:01, HLA-A*30:01, HLA-A*11:01, HLA-A*32:01, HLA-A*68:01, HLA-A*30:02, HLA-B*08:01, HLA-B*15:01, HLA-B*07:02, HLA-A*23:01 | **7L1C** |
| *** The bold font identified the Human HLA and the corresponding ID used in docking between epitopes and alleles.** | | | | | | | |

**Supplemental Table 5.** Accession numbers of 100 HIV-1 full-length sequences of the most predominate subtypes and CRFs

| **Accession Number** | **Subtype** | **Accession Number** | **Subtype** | **Accession Number** | **Subtype** |
| --- | --- | --- | --- | --- | --- |
| OQ121909.1 | G | OM209609.1 | B | MG760373.1 | AE |
| KX389646.1 | G | OM209714.1 | B | ON959789.1 | AE |
| KX228801.1 | G | OM209814.1 | B | OQ121842.1 | AG |
| JX245015.1 | O | OM209914.1 | B | MT417738.1 | AG |
| MH705150.1 | O | OM209937.1 | B | MH078539.1 | AG |
| AB703612.1 | 35-AD | OM209981.1 | B | KC473840.1 | 47-BF1 |
| OK649293.1 | C | MW881703.1 | B | MN090742.1 | B |
| OM825586.2 | C | MW881698.1 | B | MT222952.1 | B |
| OM825656.2 | C | JF320241.1 | B | MT417772.1 | B |
| OM825584.2 | C | JN692480.1 | B | ON421478.1 | B |
| AB254150.1 | C | JN692460.1 | B | ON421466.1 | B |
| AF443089.1 | C | JN692466.1 | B | ON421472.1 | B |
| AY463217.1 | C | JF320244.1 | B | MW881676.1 | B |
| AY253303.1 | C | JF320242.1 | B | MT417771.1 | B |
| OK649295.1 | C | OL446078.1 | B | MT417753.1 | B |
| OM825577.2 | C | MT559048.1 | B | ON421463.1 | B |
| AY713417.1 | C | MT559060.1 | B | ON421469.1 | B |
| AY772691.1 | C | OK514771.1 | B | MT417766.1 | B |
| AY878071.1 | C | MT559049.1 | B | MW881735.1 | B |
| DQ369982.1 | C | KR914676.1 | B | MN090736.1 | B |
| OM825578.2 | C | MZ642263.1 | A | MW262777.1 | B |
| MG365764.1 | F | MZ642262.1 | A | MW262770.1 | B |
| OM825109.2 | D | MZ642260.1 | A | ON816965.1 | B |
| OM825094.2 | D | MZ642266.1 | A | ON816970.1 | B |
| OM825104.2 | D | MZ642269.1 | A | OM209984.1 | B |
| MZ427710.1 | A6 | MT635148 | AE | MW881700.1 | B |
| MZ427733.1 | A6 | MT624753.1 | AE | MW881704.1 | B |
| OQ513524.1 | BC | AB253682.1 | AE | MW881706.1 | B |
| KF835547.1 | BC | ON902298.1 | AE | MW881707.1 | B |
| OQ092464.1 | B | MH327750.1 | AE | OM209730.1 | B |
| OQ092463.1 | B | ON816969.1 | B | OM209651.1 | B |
| OQ092465.1 | B | MW262776.1 | B | OM209404.1 | B |
| OQ092466.1 | B | OL519774.1 | B | OQ092462.1 | B |
| OQ092467.1 | B |  |  |  |  |

**Supplemental Table 6.** The consensus sequences of 11 genes of HIV-1 and vaccine construct

|  | **Consensus of 100 sequences** |
| --- | --- |
| **Gag** | MGARASVLSGGKLDRWEKIRLRPGGKKKYRLKHIVWASRELERFAVNPGLLETSEGCRQILGQLQPALQTGSEELKSLYNTVATLYCVHQRIDVKDTKEALDKIEEEQNKSKKKAQQAAADTGNSSQVSQNYPIVQNLQGQMVHQAISPRTLNAWVKVIEEKAFSPEVIPMFSALSEGATPQDLNTMLNTVGGHQAAMQMLKETINEEAAEWDRLHPVHAGPIAPGQMREPRGSDIAGTTSTLQEQIGWMTSNPPIPVGEIYKRWIILGLNKIVRMYSPVSILDIRQGPKEPFRDYVDRFYKTLRAEQATQEVKNWMTETLLVQNANPDCKTILKALGPGATLEEMMTACQGVGGPGHKARVLAEAMSQVTNSNTIMMQRGNFRNQRKTVKCFNCGKEGHIARNCRAPRKKGCWKCGKEGHQMKDCTERQANFLGKIWPSHKGRPGNFLQSRPEPTAPPESFRFGEETTTPSQKQEPIDKELYPLASLKSLFGNDPSSQ |
| **Pro** | PQITLWQRPLVTIKIGGQLKEALLDTGADDTVLEEMNLPGKWKPKMIGGIGGFIKVRQYDQIPIEICGHKAIGTVLVGPTPVNIIGRNLLTQIGCTLNF |
| **RT** | EICGHKAIGTVLVGPTPVNIIGRNLLTQIGCTLNFPISPIETVPVKLKPGMDGPKVKQWPLTEEKIKALVEICTEMEKEGKISKIGPENPYNTPVFAIKKKDSTKWRKLVDFRELNKRTQDFWEVQLGIPHPAGLKKKKSVTVLDVGDAYFSVPLDEDFRKYTAFTIPSINNETPGIRYQYNVLPQGWKGSPAIFQSSMTKILEPFRKQNPDIVIYQYMDDLYVGSDLEIGQHRTKIEELRQHLLKWGFTTPDKKHQKEPPFLWMGYELHPDKWTVQPIVLPEKDSWTVNDIQKLVGKLNWASQIYPGIKVKQLCKLLRGTKALTEVVPLTEEAELELAENREILK |
| **IN** | FLDGIDKAQEEHEKYHSNWRAMASDFNLPPVVAKEIVASCDKCQLKGEAMHGQVDCSPGIWQLDCTHLEGKIILVAVHVASGYIEAEVIPAETGQETAYFILKLAGRWPVKTIHTDNGSNFTSTTVKAACWWAGIKQEFGIPYNPQSQGVVESMNKELKKIIGQVRDQAEHLKTAVQMAVFIHNFKRKGGIGGYSAGERIIDIIATDIQTKELQKQITKIQNFRVYYRDSRDPLWKGPAKLLWKGEGAVVIQDNSDIKVVPRRKAKIIRDYGKQMAGDDCVASRQDED |
| **Vif** | MENRWQVMIVWQVDRMRIRTWKSLVKHHMYVSKKAKGWFYRHHYESRHPRISSEVHIPLGDARLVITTYWGLHTGERDWHLGQGVSIEWRKRRYSTQVDPDLADQLIHLYYFDCFSESAIRKAILGHIVSPRCEYQAGHNKVGSLQYLALTALITPKKIKPPLPSVRKLTEDRWNKPQKTKGHRGSHTMNGH |
| **Vpr** | MEQAPEDQGPQREPYNEWTLELLEELKNEAVRHFPRPWLHGLGQYIYETYGDTWAGVEAIIRILQQLLFIHFRIGCQHSRIGIIRQRRARNGASRS |
| **Tat** | MEPVDPRLEPWKHPGSQPKTACTKCYCKKCCFHCQVCFLTKGLGISYGRKKRRQRRRAPQSSQDHQVPLSKQPLSQPRGDPTGPKESKKKVERETETDPD |
| **Rev** | MAGRSGDSDEELLKAVRIIKILYQSNPPPSPEGTRQARRNRRRRWRERQRQIRSISERILSTCLGRPAEPVPLQLPPLERLTLDCSEDCGTSGTQGVGSPQILVESPAVLESGTKE |
| **Vpu** | MQPLQILAIVALVVAAIIAIVVWTIVFIEYRKILRQRKIDRLIDRIRERAEDSGNESEGDQEELSALVEMGHHAPWDVDDL |
| **Env** | MRVKGIRKNYQHLWRWGILLLGMLMICSATENLWVTVYYGVPVWKEATTTLFCASDAKAYDTEVHNVWATHACVPTDPNPQEVVLENVTENFNMWKNNMVEQMHEDIISLWDQSLKPCVKLTPLCVTLNCTDVNNTTNSNKGEIKNCSFNITTEIRDKVQKEYALFYKLDIVPIDNDNNSYRLINCNTSVITQACPKVSFEPIPIHYCAPAGFAILKCNDKTFNGTGPCKNVSTVQCTHGIKPVVSTQLLLNGSLAEEEIVIRSENFTDNAKTIIVQLNESVEINCTRPNNNTRKSIHIGPGRAFYATGDIIGDIRQAHCNISKTKWNNTLQQVAKKLREQFGNKTIVFNPSSGGDPEITMHSFNCGGEFFYCNTTQLFNSTWNNETITLPCRIKQIINMWQEVGKAMYAPPIRGNITCSSNITGLLLTRDGGNNNNSNTETFRPGGGDMRDNWRSELYKYKVVKIEPLGVAPTKAKRRVVQREKRAVGLGAVFLGFLGAAGSTMGAASITLTVQARQLLSGIVQQQNNLLRAIEAQQHLLQLTVWGIKQLQARVLAVERYLKDQQLLGIWGCSGKLICTTTVPWNSSWSNKSLDEIWDNMTWMQWEREIDNYTGLIYTLLEESQNQQEKNEQELLELDKWASLWNWFDITNWLWYIKIFIMIVGGLIGLRIVFAVLSIVNRVRQGYSPLSFQTLLPAPRGPDRPEGIEEEGGERDRDRSIRLVSGFLALIWDDLRSLCLFSYHRLRDLLLIVARIVELLGRRGWEALKYWWNLLQYWSQELKNSAISLLNTTAIAVAEGTDRIIEVVQRIGRAILHIPRRIRQGLERALL |
| **Nef** | MGGKWSKSSIVGWPAVRERMRRTEPAAEGVGAASRDLEKHGAITSSNTAANNADCAWLEAQEEEEVGFPVRPQVPLRPMTYKGAFDLSHFLKEKGGLEGLIYSQKRQEILDLWVYHTQGYFPDWQNYTPGPGVRYPLTFGWCFKLVPVDPEEVEEANEGENNCLLHPMSQHGMDDPEREVLMWKFDSRLAFRHMARELHPEYYKDC |
| **Vaccine construct** | GIINTLQKYYCRVRGGRCAVLSCLPKEEQIGKCSTRGRKCCRRKKEAAKAKFVAAWTLKAAAGGGSLKHIVWASRGGGSTIKIGGQLKGGGSNNETPGIRYGGGSETAYFILKLGGGSLQYLALTALGGGSDTWAGVEAIGGGSGLGISYGRKGGGSGRPAEPVPLGGGSTIVFIEYRKGGGSGLRIVFAVLGGGSRLAFRHMARGPGPGGGKLDRWEKIRLRPGGPGPGPGKWKPKMIGGIGGFGPGPGDFRKYTAFTIPSINNGPGPGDFNLPPVVAKEIVASGPGPGGVSIEWRKRRYSTQVGPGPGLGISYGRKKRRQRRGPGPGTIVFIEYRKILRQRKGPGPGPIHYCAPAGFAILKCGPGPGVGWPAVRERMRRTEPKKIRLRPGGKKIKIGGQLKKIPSINNEKKSGYIEAEKKSIEWRKRKKCQHSRIGKKTKGLGISKKPAEPVPLKKIDRIRERKKHYCAPAGKKMRRTEPAEGGETAKSKKFPSYTATYQF |

**Supplemental Table 7.** A list of conserved domains of all gene sequences of HIV-1

| **Genes Name** | **Conserved Domain(s)** | **Description** | **Interval** |
| --- | --- | --- | --- |
| **Gag**  **ORF** | Gag_p17 | Gag gene protein p17 (matrix protein): The matrix protein forms an icosahedral shell associated with the inner membrane of the mature immunodeficiency virus. | 2-132 |
|  | Gag_p24_C | Gag protein p24 C-terminal domain; p24 forms the inner protein layer of the nucleocapsid. ELISA tests for p24 are the most commonly used method to demonstrate virus replication both in vivo and in vitro. | 278-349 |
|  | Gag_p6 | Gag protein p6; HIV protein p6 contains two late-budding domains (L domains), which are short sequence motifs essential for viral particle release. p6 interacts with the endosomal sorting complex and represents a docking site for several cellular and binding factors. The PTAP motif interacts with the cellular budding factor TSG101. This domain is also found in some chimpanzee immunodeficiency virus (SIV-cpz) proteins. | 449-485 |
|  | PTZ00368 superfamily | Universal minicircle sequence binding protein (UMSBP); Provisional | 349-429 |
|  | Gag_p24 | Gag gene protein p24 (core nucleocapsid protein); p24 forms the inner protein layer of the nucleocapsid. ELISA tests for p24 are the most commonly used method to demonstrate virus replication both in vivo and in vitro. | 143-268 |
| **Pro** | pepsin_retropepsin_like superfamily | Cellular and retroviral pepsin-like aspartate proteases: This family includes both cellular ... | 5-98 |
| **RT** | RT_Rtv | RT_Rtv: Reverse transcriptases (RTs) from retroviruses (Rtvs). RTs catalyze the conversion of ... | 53-269 |
|  | RVT_thumb superfamily | Reverse transcriptase thumb domain: This domain is known as the thumb domain. It is composed ... | 276-331 |
|  | pepsin_retropepsin_like superfamily | Cellular and retroviral pepsin-like aspartate proteases: This family includes both cellular ... | 1-34 |
| **IN** | rve superfamily | Integrase core domain: Integrase mediates integration of a DNA copy of the viral genome into ... | 57-145 |
|  | IN_DBD_C superfamily | Integrase DNA binding domain: Integrase mediates integration of a DNA copy of the viral genome ... | 225-268 |
|  | Integrase_Zn superfamily | Integrase Zinc binding domain; Integrase mediates integration of a DNA copy of the viral ... | 10-44 |
| **Vif** | Vif superfamily | Retroviral Vif (Viral infectivity) protein; Human immunodeficiency virus type 1 (HIV-1) Vif is ... | 1-192 |
| **Vpr** | VPR superfamily | VPR/VPX protein; | 1-83 |
| **Tat** | Tat superfamily | Transactivating regulatory protein (Tat): The retroviral Tat protein binds to the Tar RNA. ... | 2-63 |
| **Rev** | REV superfamily | REV protein (anti-repression trans-activator protein); | 1-91 |
| **Vpu** | Vpu superfamily | Vpu protein: The Vpu protein contains an N-terminal transmembrane spanning region and a ... | 23-78 |
| **Env** | GP120 superfamily | Envelope glycoprotein GP120: The entry of HIV requires the interaction of viral GP120 with CD4 and ... | 33-486 |
|  | Ebola_HIV-1-like_HR1-HR2 superfamily | Heptad repeat 1-heptad repeat two regions (ectodomain) of the transmembrane subunit of various ... | 505-695 |
| **Nef** | F-protein | Negative factor (F-Protein) or Nef; Nef protein accelerates virulent progression of AIDS by ... | 2-205 |

**Supplemental Table 8.** ProtParam results of the eleven HIV polyproteins, proteins, and vaccine construct.

|  | **GAG** | **PRO** | **RT** | **INT** | **VIF** | **VPR** | **TAT** | **REV** | **VPU** | **ENV** | **NEF** | **Vaccine**  **construct** |
| --- | --- | --- | --- | --- | --- | --- | --- | --- | --- | --- | --- | --- |
| **Instability index** | 44.88 | 34.21 | 36.28 | 35.88 | 41.16 | 39.37 | 65.81 | 101.33 | 34.27 | 42.54 | 47.21 | 42.44 |
| **Aliphatic index** | 70.60 | 115.15 | 90.92 | 83.99 | 77.14 | 85.42 | 37.00 | 80.69 | 130.00 | 93.33 | 62.04 | 66.56 |
| **GRAVY** | -0.644 | 0.133 | -0.438 | -0.401 | -0.701 | -0.662 | -1.333 | -0.762 | 0.101 | -0.256 | -0.677 | -0.582 |
| **Number of amino acids** | 499 | 99 | 346 | 288 | 192 | 96 | 100 | 116 | 81 | 871 | 206 | 503 |
| **Molecular weight** | 55743.73 | 10775.76 | 39581.03 | 32240.85 | 22728.21 | 11356.87 | 11478.19 | 12931.69 | 9291.74 | 98958.42 | 23618.58 | 53436.29 |
| **Theoretical pI** | 9.27 | 8.81 | 8.88 | 7.75 | 10.07 | 6.91 | 9.61 | 9.41 | 4.72 | 8.31 | 5.30 | 10.70 |
| **Half-life** | | | | | | | | | | | | |
| **Mammalian reticulocytes (**hours) | 30 hours | >20 hours | 1 hour | 1.1 hours | 30 hours | 30 hours | 30 hours | 30 hours | 30 hours | 30 hours | 30 hours | 30 hours |
| **Yeast (**hours) | >20 hours | >20 hours | 30 min | 3 min | >20 hours | >20 hours | >20 hours | >20 hours | >20 hours | >20 hours | >20 hours | >20 hours |
| **Escherichia coli (**hours) | >10 hours | >10 hours | >10 hours | 2 min | >10 hours | >10 hours | >10 hours | >10 hours | >10 hours | >10 hours | >10 hours | >10 hours |

**Supplemental Table 9.** Numbers of predicted B cell epitopes

| **Gene Name** | **Primary epitopes** | **Final epitopes** |
| --- | --- | --- |
| **Gag** | ABCpred=50, Bepipred=11, Emini= 494, Karplus=492, Parker=493  Total= 1545 | 78 |
| **Pro** | ABCpred=9, Bepipred=4, Emini= 94, Karplus=92, Parker=93  Total= 292 | 9 |
| **RT** | ABCpred=38, Bepipred=16, Emini= 349, Karplus=339, Parker=340  Total= 1082 | 76 |
| **IN** | ABCpred=28, Bepipred=11, Emini= 289, Karplus=282, Parker=282  Total= 893 | 36 |
| **Vif** | ABCpred=22, Bepipred=5, Emini= 191, Karplus=185, Parker=186  Total= 589 | 34 |
| **Vpr** | ABCpred=7, Bepipred=4, Emini= 93, Karplus=89, Parker=92  Total= 285 | 13 |
| **Tat** | ABCpred=7, Bepipred=2, Emini= 95, Karplus=95, Parker=96  Total= 295 | 13 |
| **Rev** | ABCpred=10, Bepipred=3, Emini= 111, Karplus=109, Parker=110  Total= 343 | 19 |
| **Vpu** | ABCpred=4, Bepipred=2, Emini= 76, Karplus=76, Parker=75  Total= 233 | 14 |
| **Env** | ABCpred=88, Bepipred=27, Emini= 846, Karplus=824, Parker=825  Total= 2610 | 155 |
| **Nef** | ABCpred=15, Bepipred=9, Emini= 201, Karplus=199, Parker=199  Total= 423 | 54 |
| **All** | **8590** | **488** |

**Supplemental Table 10.** Numbers of predicted HTL epitopes

| **Gene Name** | **Primary epitopes** | **Final epitopes (common between humans and mouse*)*** |
| --- | --- | --- |
| Gag | 3888 | 6 |
| Pro | 1593 | 0 |
| RT | 1593 | 2 |
| IN | 4105 | 11 |
| Vif | 2107 | 4 |
| Vpr | 1107 | 2 |
| Tat | 378 | 4 |
| Rev | 1080 | 0 |
| Vpu | 621 | 1 |
| Env | 3700 | 8 |
| Nef | 837 | 6 |
| **Total** | **21009** | **44** |

**Supplemental Table 11.** Numbers of predicted CTL epitopes

| **Gene Name** | **Primary epitopes** | **Final epitopes (common between human and mouse)** |
| --- | --- | --- |
| **Gag** | 5892 | 14 |
| **Pro** | 1092 | 1 |
| **RT** | 4056 | 17 |
| **IN** | 3360 | 11 |
| **Vif** | 2208 | 13 |
| **Vpr** | 1056 | 4 |
| **Tat** | 1104 | 2 |
| **Rev** | 1296 | 5 |
| **Vpu** | 876 | 14 |
| **Env** | 9876 | 69 |
| **Nef** | 2376 | 12 |
| **Total** | **33192** | **162** |

**Supplemental Table 12.** The epitopes of the vaccine construct were confirmed by comparing the sequences obtained from two databases, NCBI and ENA.

| **Similarity of LANL epitopes with ENA seqs** | | **Similarity of LANL epitopes with NCBI seqs** | | **Epitopes predicted using LANL seqs** |
| --- | --- | --- | --- | --- |
| With few mismatches | 100% identical | With few mismatches | 100% identical |  |
| 2/11 (18.18%) | 9/11 (81.81%) | 3/11 (27.27%) | 8/11 (72.7%) | B cell epitopes |
| 5/9 (55.55%) | 4/9 (44.44%) | 4/9 (44.44%) | 5/9 (55.5%) | HLA epitopes |
| 4/11 (36.36%) | 7/11 (63.63%) | 3/11 (27.27%) | 8/11 (72.7%) | CD8 epitopes |

**Supplemental Table 13.** Population coverage results of selected CTL and HTL epitopes

| **MHC Class I/II** | **Coverage** | **Average-hit** | **PC90** |
| --- | --- | --- | --- |
| Combined | 95.04 | 14.2 | 6.93 |

**Supplemental Table 14.** Vaccine sequence construct properties. The sequence of the vaccine model was antigenic, non-toxic, non-allergenic, non-homologous to human proteome, soluble, and without any signal sequence.

|  | | **Vaccine** |
| --- | --- | --- |
| **Toxin** | | Non-Toxin |
| **Allergenicity** | | NON-ALLERGEN |
| **VaxiJen** | | 0.7739 (Probable Antigen) |
| **Topology** | | Inside |
| **Homology** | | Nonhomologous to human |
| **Solubility** | **Predicted scaled solubility** | 0.645 |
|  | **pI** | 11.330 |

**Supplemental Table 15.** Various features of the secondary structures in HIV genes and vaccine construct

|  | **9a** | | | | | | | | | | | **9b** |
| --- | --- | --- | --- | --- | --- | --- | --- | --- | --- | --- | --- | --- |
|  | **Gag** | **Pro** | **RT** | **IN** | **Vif** | **Vpr** | **Tat** | **Rev** | **Vpu** | **Env** | **Nef** | **Vaccine**  **construct** |
| **Alpha helix** | 45.49% | 8.08% | 32.37% | 40.97% | 33.33% | 51.4% | 8.00% | 35.34% | 72.84% | 44.89% | 36.41% | 14.12% |
| **Extended strand** | 7.62% | 43.43% | 19.65% | 16.67% | 17.71% | 10.42% | 10.00% | 8.62% | 4.94% | 19.25% | 11.17% | 25.25% |
| **Beta turn** | 4.41% | 7.07% | 7.23% | 6.94% | 1.56% | 4.17% | 3.00% | 0.00% | 3.70% | 3.85% | 5.34% | 8.75% |
| **Random coil** | 42.48% | 41.41% | 40.75% | 35.42% | 47.40% | 34.38% | 79.00% | 56.03% | 18.52% | 32.01% | 47.09% | 51.89% |

**Supplemental Table 16.** Results of validation and determination of the best model of full vaccine using I-TASSER, Robetta, and AlphaFold servers

| **Best Model** | **ProSAZ-Score** | **ERRAT-A** | **QMEAN** | **Favored region** | **Allowed region** |
| --- | --- | --- | --- | --- | --- |
| **I-TASSER** | -7.29 | 89.32 | -2.9 | 79.90% | 18.40% |
| **Robetta** | -4.27 | 74.43 | -15.42 | 53.60% | 41.70% |
| **AlphaFold** | -3.76 | 70.11 | -12.66 | 71.90% | 27.00% |

**Supplemental Table 17.** Disulfide engineering results of the vaccine construct using by in the DbD2 online server

| **Res1 Chain** | **Res1 Seq #** | **Res1 AA** | **Res2 Chain** | **Res2 Seq #** | **Res2 AA** | **Chi3** | **Energy** | **Sum**  **B-Factors** |
| --- | --- | --- | --- | --- | --- | --- | --- | --- |
| A | 8 | LYS | A | 24 | LEU | -115.21 | 5.02 | 8.99 |
| A | 10 | TYR | A | 39 | LYS | -96.66 | 4.5 | 10.11 |
| A | 11 | CYS | A | 18 | CYS | 90.89 | 3.79 | 8.6 |
| A | 22 | SER | A | 29 | GLN | 69.83 | 7.03 | 9.6 |
| ***A*** | ***45*** | ***LYS*** | ***A*** | ***48*** | ***ALA*** | ***118.81*** | ***1.77*** | ***9.41*** |
| A | 72 | TRP | A | 76 | GLY | 68.46 | 4.11 | 8.82 |
| A | 77 | GLY | A | 116 | GLY | -64.99 | 6.8 | 8.74 |
| A | 105 | SER | A | 124 | LEU | 97.55 | 3.05 | 9.89 |
| A | 114 | LEU | A | 118 | SER | 111.32 | 5.71 | 8.24 |
| A | 129 | GLY | A | 235 | TYR | -73.67 | 4.52 | 11.91 |
| A | 135 | ALA | A | 144 | SER | 81.69 | 3.48 | 16.83 |
| A | 135 | ALA | A | 146 | LEU | 67.25 | 2.61 | 16.66 |
| A | 149 | SER | A | 151 | GLY | 100.49 | 3.82 | 15.42 |
| A | 154 | GLY | A | 237 | ALA | 117.23 | 5.01 | 9.24 |
| A | 155 | GLY | A | 238 | PHE | 102.33 | 2.37 | 8.41 |
| A | 158 | GLY | A | 162 | GLU | 99.28 | 3.23 | 8.69 |
| A | 158 | GLY | A | 164 | VAL | 118.61 | 6.67 | 8.51 |
| A | 160 | PRO | A | 243 | ILE | 115.55 | 2.96 | 8.81 |
| A | 161 | ALA | A | 245 | ASN | -99.93 | 2.62 | 8.92 |
| A | 163 | PRO | A | 216 | ARG | -69.27 | 3.9 | 10.21 |
| ***A*** | ***163*** | ***PRO*** | ***A*** | ***218*** | ***GLU*** | ***92.99*** | ***1.92*** | ***8.94*** |
| A | 176 | GLU | A | 229 | PRO | -110.99 | 3.24 | 11.36 |
| A | 177 | TYR | A | 188 | VAL | -60.05 | 4.14 | 10.07 |
| A | 179 | LYS | A | 227 | PRO | -113.35 | 3.76 | 9.3 |
| ***A*** | ***179*** | ***LYS*** | ***A*** | ***230*** | ***GLY*** | ***111.18*** | ***1.81*** | ***9.84*** |
| A | 200 | PHE | A | 203 | MET | 90.52 | 3.53 | 14.23 |
| A | 216 | ARG | A | 218 | GLU | 106.99 | 2.41 | 10.45 |
| ***A*** | ***220*** | ***ILE*** | ***A*** | ***242*** | ***SER*** | ***-77.66*** | ***0.55*** | ***8.71*** |
| A | 221 | ARG | A | 224 | PRO | 101.66 | 3.46 | 8.68 |
| A | 228 | GLY | A | 237 | ALA | -70.49 | 4.92 | 8.95 |
| ***A*** | ***231*** | ***ASP*** | ***A*** | ***234*** | ***LYS*** | ***117.71*** | ***2.11*** | ***10.34*** |
| A | 261 | GLU | A | 262 | ILE | -105.77 | 2.81 | 8.9 |
| A | 261 | GLU | A | 344 | GLU | 92.1 | 5.3 | 8.16 |
| ***A*** | ***266*** | ***GLY*** | ***A*** | ***314*** | ***TYR*** | ***111.95*** | ***1.03*** | ***12.82*** |
| A | 268 | GLY | A | 313 | HIS | 126.61 | 5.66 | 13.1 |
| A | 276 | TRP | A | 292 | ILE | 124.83 | 3.42 | 8.5 |
| A | 283 | THR | A | 286 | GLY | 121.5 | 3.6 | 8.32 |
| A | 319 | GLY | A | 350 | LEU | 88.84 | 3.64 | 10.01 |
| A | 352 | PRO | A | 446 | GLY | -78.96 | 7.33 | 8.41 |
| A | 353 | GLY | A | 354 | GLY | 114.57 | 4.92 | 8.24 |
| A | 363 | LEU | A | 419 | LYS | 114.07 | 3.74 | 8.71 |
| A | 365 | LYS | A | 417 | LEU | 117.2 | 7.23 | 10.21 |
| A | 368 | SER | A | 415 | VAL | -99.89 | 6.81 | 8.5 |
| A | 370 | ASN | A | 413 | GLU | 98.17 | 6.62 | 8.43 |
| A | 383 | LYS | A | 434 | ALA | 74.9 | 7.97 | 12.8 |
| A | 384 | SER | A | 397 | ARG | 84.6 | 5.36 | 13.37 |
| A | 385 | ILE | A | 386 | GLU | -115.52 | 2.97 | 12.58 |
| A | 403 | LYS | A | 420 | ILE | -101.93 | 5.22 | 8.91 |
| A | 408 | SER | A | 412 | ALA | -83.54 | 5.91 | 8.46 |
| A | 434 | ALA | A | 442 | GLU | -68.97 | 7.15 | 10.13 |
| A | 435 | GLY | A | 441 | THR | -102.62 | 4.06 | 8.72 |
| A | 444 | ALA | A | 447 | GLY | -112.72 | 3.89 | 8.58 |
| A | 452 | SER | A | 464 | PHE | 99.19 | 3.07 | 9.02 |

**Supplemental Table 18.** The data of residues and scores of predicted discontinuous B-cell epitopes on vaccine construct.

| **No.** | **Residues** | **Number of residues** | **Score** |
| --- | --- | --- | --- |
| **1** | A:G1, A:I2, A:I3, A:N4, A:T5, A:L6, A:Q7, A:K8, A:Y9, A:Y10, A:C11, A:R12, A:V13, A:G15, A:G16, A:R17, A:C18, A:A19, A:V20, A:L21, A:S22, A:C23, A:L24, A:P25, A:K26, A:E27, A:E28, A:Q29, A:I30, A:G31, A:K32, A:C33, A:S34, A:T35, A:R36, A:G37, A:R38, A:K39, A:C40, A:C41, A:R42, A:R43, A:K44, A:K45, A:E46, A:A47, A:A48, A:K49, A:A50, A:K51, A:F52, A:V53, A:A54, A:A55, A:W56, A:T57, A:L58, A:W72, A:A73, A:S74, A:R75, A:G76, A:G77, A:G78, A:S79, A:T80, A:I81, A:S92, A:N93, A:N94, A:E95, A:T96, A:K113, A:L114, A:G115, A:G116, A:G117, A:S118, A:L119 | 79 | 0.771 |
| **2** | A:F455, A:P456, A:S457 | 3 | 0.718 |
| **3** | A:P345, A:K346, A:K347, A:K355, A:K356, A:I357, A:K358, A:I359, A:G360, A:G361, A:Q362, A:L363, A:K364, A:I369, A:N370, A:N371, A:E372, A:K373, A:K374, A:S375, A:G376, A:Y377, A:I378, A:E379, A:A380, A:E381, A:K382, A:K383, A:S384, A:I385, A:E386, A:R388, A:K389, A:K392, A:C393, A:Q394, A:H395, A:S396, A:R397, A:I398, A:G399, A:K400, A:K401, A:T402, A:K403, A:G404, A:L405, A:G406, A:I407, A:S408, A:K409, A:K410, A:P411, A:A412, A:E413, A:P414, A:V415, A:P416, A:L417, A:K418, A:K419, A:I420, A:D421, A:R422, A:I423, A:R424, A:E425, A:R426, A:K427, A:K428, A:H429, A:Y430, A:C431, A:A432, A:P433, A:A434, A:G435, A:K436, A:K437, A:R440, A:T441, A:E442, A:P443, A:A444 | 84 | 0.703 |
| **4** | A:P160, A:A161, A:E162, A:P163, A:V164, A:G169, A:E176, A:G180, A:G181, A:G182, A:S183, A:G184, A:L185, A:R186, A:I187, A:V188, A:F189, A:A190, A:V191, A:L192, A:G193, A:G194, A:G195, A:S196, A:R197, A:L198, A:A199, A:F200, A:R201, A:H202, A:M203, A:A204, A:R205, A:G206, A:P207, A:G208, A:P209, A:G210, A:G211, A:G212, A:K213, A:L214, A:D215, A:R216, A:W217, A:E218, A:K219, A:I220, A:R221, A:P224, A:I243, A:N244, A:N245, A:G246, A:P247, A:G248, A:P249, A:G250, A:D251 | 59 | 0.646 |
| **5** | A:S452, A:K453, A:Y458, A:T459, A:A460, A:T461 | 6 | 0.57 |
| **6** | A:F252, A:A264, A:S265, A:G266, A:P267, A:G268, A:P269, A:G270, A:G271, A:V272, A:S273, A:I274, A:E275, A:W276, A:L301, A:R302, A:Q303, A:R304, A:K305, A:G306, A:P307, A:G308, A:P309, A:G310, A:P311, A:I312, A:H313, A:Y314, A:G326, A:P327, A:G328, A:P329, A:G330, A:V331, A:G332 | 35 | 0.536 |

**Supplemental Table 19.** The data of residues and scores of predicted linear B-cell epitopes on vaccine construct.

| **No.** | **Chain** | **Start** | **End** | **Peptide** | **Number of residues** | **Score** |
| --- | --- | --- | --- | --- | --- | --- |
| **1** | A | 1 | 58 | GIINTLQKYYCRVRGGRCAVLSCLPKEEQIGKCSTRGRKCCRRKKEAAKAKFVAAWTL | 58 | 0.848 |
| **2** | A | 369 | 442 | INNEKKSGYIEAEKKSIEWRKRKKCQHSRIGKKTKGLGISKKPAEPVPLKKIDRIRERKKHYCAPAGKKMRRTE | 74 | 0.728 |
| **3** | A | 356 | 364 | KIKIGGQLK | 9 | 0.717 |
| **4** | A | 180 | 224 | GGGSGLRIVFAVLGGGSRLAFRHMARGPGPGGGKLDRWEKIRLRP | 45 | 0.682 |
| **5** | A | 301 | 313 | LRQRKGPGPGPIH | 13 | 0.652 |
| **6** | A | 72 | 81 | WASRGGGSTI | 10 | 0.629 |
| **7** | A | 243 | 250 | INNGPGPG | 8 | 0.617 |
| **8** | A | 452 | 464 | SKKFPSYTATYQF | 13 | 0.586 |
| **9** | A | 114 | 119 | LGGGSL | 6 | 0.582 |
| **10** | A | 270 | 275 | GGVSIE | 6 | 0.56 |

**Supplemental Table 20.** Molecular docking results of TLRs and Vaccine construct using Cluspro2.0 tool. (The best models are marked with a green box)

|  | **Lowest energy scores** | | |
| --- | --- | --- | --- |
| **Predicted Models** | **Vaccine + TLR3** | **Vaccine + TLR4** | **Vaccine + TLR9** |
| **0** | -1178.2 | -1111.9 | -1251.7 |
| **1** | -1155.5 | -1375.0 | -1231.9 |
| **2** | -1079.7 | -1381.5 | -1355.9 |
| **3** | -1284.4 | -1173.1 | -1183.4 |
| **4** | -1089.0 | -1229.5 | -1137.6 |
| **5** | -1158.4 | -1269.3 | -1168.6 |
| **6** | -1035.2 | -1184.0 | -1246.4 |
| **7** | -1181.2 | -1225.5 | -1264.4 |
| **8** | -1119.1 | -1130.5 | -1102.1 |
| **9** | -1120.7 | -1079.4 | -1171.1 |
| **10** | -1106.8 | -1308.9 | -1161.9 |
| **11** | -1092.5 | -1138.4 | -1149.2 |
| **12** | -1069.4 | -1121.2 | -1190.3 |
| **13** | -1228.1 | -1119.6 | -1074.6 |
| **14** | -1178.7 | -1056.9 | -1233.1 |
| **15** | -1023.3 | -1247.5 | -1215.4 |
| **16** | -1033.7 | -1114.9 | -1051.4 |
| **17** | -1039.2 | -1156.1 | -1147.1 |
| **18** | -1123.6 | -1152.3 | -1050.9 |
| **19** | -1040.1 | -1141.2 | -1115.9 |
| **20** | -1063.1 | -1187.4 | -1168.0 |
| **21** | -1022.6 | -1133.0 | -1137.3 |
| **22** | -1020.1 | -1141.8 | -1100.4 |
| **23** | -1289.8 | -1110.6 | -1028.7 |
| **24** | -1024.7 | -1081.1 | -1163.6 |
| **25** | -1000.1 | -1035.0 | -1116.8 |
| **26** | -987.9 | -1173.3 | -1052.5 |
| **27** | -969.8 | -1156.6 | -1034.0 |
| **28** | -1068.3 | -1272.1 | -1067.9 |
| **29** | -1086.6 | -1060.8 | -1171.8 |

**Supplemental Table 21.** The sequence of the optimized codon of the vaccine models in human and E. coli hosts.

| **Hosts** | **Codon optimized sequences** |
| --- | --- |
| **Human** | ATGGGAATTATCAATACACTGCAGAAGTACTATTGCAGGGTCAGAGGTGGCAGATGCGCAGTGCTGTCTTGTCTGCCCAAGGAGGAACAGATTGGCAAGTGCTCAACCAGGGGCAGAAAATGTTGTCGCAGGAAGAAGGAGGCCGCTAAGGCCAAATTCGTCGCCGCCTGGACCCTGAAAGCAGCTGCCGGTGGGGGCTCCCTGAAACATATCGTGTGGGCCTCACGGGGCGGAGGCTCCACTATCAAAATCGGCGGACAGCTGAAGGGCGGCGGGAGCAATAACGAGACCCCAGGCATCAGATACGGCGGCGGAAGCGAGACTGCCTACTTCATCCTGAAGCTGGGAGGCGGCTCCCTGCAGTATCTGGCCCTGACAGCTCTGGGGGGGGGTTCCGATACTTGGGCCGGTGTGGAGGCCATCGGAGGGGGCAGCGGACTGGGAATCAGTTACGGAAGGAAGGGGGGCGGCAGCGGGCGGCCCGCAGAGCCCGTGCCCCTGGGCGGAGGCTCCACTATTGTGTTCATCGAGTATAGAAAGGGCGGCGGCTCCGGGCTGAGGATCGTGTTCGCTGTGCTGGGAGGAGGATCCCGGCTGGCTTTTAGGCACATGGCCCGCGGCCCAGGCCCAGGAGGCGGGAAACTGGACCGGTGGGAGAAGATCAGGCTGAGACCCGGAGGGCCCGGCCCCGGGCCAGGGAAGTGGAAGCCTAAGATGATCGGAGGGATTGGAGGCTTTGGCCCAGGGCCCGGAGATTTTAGAAAATACACAGCATTCACAATCCCTTCCATCAATAACGGCCCTGGCCCCGGCGACTTTAACCTGCCACCTGTGGTGGCAAAAGAGATCGTGGCCAGTGGCCCCGGCCCTGGCGGGGTTAGTATCGAGTGGCGGAAGAGAAGGTACTCCACCCAGGTGGGCCCCGGGCCCGGACTGGGCATCTCCTACGGCAGAAAGAAGCGGAGACAGCGGCGGGGCCCAGGACCCGGCACAATCGTGTTTATCGAGTACAGGAAGATCCTGAGACAGAGGAAAGGGCCCGGCCCCGGCCCAATTCACTATTGTGCTCCTGCCGGCTTCGCTATTCTCAAGTGCGGCCCCGGCCCCGGCGTGGGATGGCCAGCTGTGAGGGAGAGAATGAGACGCACCGAGCCCAAGAAGATCCGGCTGAGGCCAGGAGGCAAGAAGATCAAGATTGGCGGCCAGCTGAAGAAGATCCCATCCATTAATAACGAAAAAAAATCTGGCTACATCGAGGCCGAAAAGAAATCCATCGAGTGGAGAAAGAGGAAAAAATGCCAGCACTCACGCATCGGCAAGAAAACCAAAGGCCTGGGGATCTCCAAGAAACCAGCCGAGCCTGTCCCACTTAAGAAGATCGATAGGATTAGAGAGAGAAAGAAACACTACTGCGCTCCTGCCGGAAAGAAAATGAGACGGACAGAGCCCGCCGAAGGCGGCGAGACAGCCAAGAGCAAGAAGTTCCCTTCTTATACCGCCACCTACCAGTTC |
| **E. coli** | ATGGGGATCATTAACACCCTGCAGAAATATTACTGCAGAGTGAGAGGCGGCAGATGTGCCGTCCTGAGCTGTCTGCCTAAGGAGGAGCAGATCGGAAAGTGCTCCACCCGGGGCCGCAAGTGCTGCCGGAGAAAAAAGGAGGCCGCCAAGGCCAAGTTCGTGGCCGCCTGGACCCTGAAGGCCGCTGCAGGAGGCGGCAGTCTCAAGCACATCGTGTGGGCTAGTAGGGGCGGCGGCAGCACCATCAAGATTGGTGGCCAGCTGAAGGGCGGAGGCAGCAACAACGAAACACCCGGGATCAGATATGGAGGCGGAAGCGAAACCGCCTACTTCATCCTGAAGCTCGGCGGGGGCAGCCTGCAGTATCTGGCTCTGACCGCCCTGGGAGGAGGCAGCGACACCTGGGCCGGCGTGGAAGCCATCGGCGGGGGATCTGGACTGGGAATCAGCTACGGCCGCAAAGGAGGAGGCTCTGGCAGACCTGCAGAACCTGTGCCTCTGGGCGGGGGCAGCACTATCGTGTTTATCGAGTACAGGAAGGGCGGTGGAAGCGGGCTGAGAATCGTGTTTGCCGTCCTGGGAGGCGGAAGCAGGCTGGCCTTCAGGCACATGGCCAGAGGCCCCGGCCCTGGAGGCGGGAAGCTCGACAGGTGGGAGAAGATCAGGCTGAGACCTGGCGGCCCTGGTCCTGGCCCTGGGAAGTGGAAACCTAAGATGATCGGCGGCATTGGCGGCTTCGGCCCTGGGCCTGGGGACTTCCGGAAGTACACAGCTTTCACAATCCCTTCTATTAACAACGGTCCTGGACCTGGCGATTTCAACCTGCCACCAGTGGTCGCCAAAGAGATCGTGGCTTCCGGCCCAGGCCCCGGGGGCGTGAGCATCGAGTGGCGGAAAAGAAGATACTCAACACAGGTGGGCCCTGGCCCAGGCCTGGGCATTAGCTACGGCAGGAAGAAGAGAAGGCAGAGAAGGGGACCCGGGCCAGGTACCATCGTGTTCATTGAATACCGGAAGATTCTGCGCCAGCGCAAAGGCCCCGGCCCAGGCCCTATTCATTACTGCGCCCCTGCAGGATTTGCCATCCTGAAGTGTGGCCCAGGACCTGGCGTGGGGTGGCCCGCTGTGCGCGAAAGGATGCGGAGAACTGAGCCCAAGAAGATCAGGCTGAGGCCAGGAGGCAAGAAGATTAAGATCGGAGGGCAGCTGAAGAAGATCCCATCAATCAATAATGAGAAGAAGTCCGGGTATATTGAGGCAGAGAAGAAGAGCATTGAGTGGAGAAAGAGGAAGAAGTGTCAGCACAGCCGAATTGGCAAGAAGACCAAGGGCCTGGGAATATCTAAGAAGCCAGCCGAGCCAGTGCCACTCAAGAAGATCGACAGAATTAGGGAGAGGAAGAAGCACTACTGCGCCCCCGCAGGAAAGAAGATGCGAAGGACAGAGCCAGCAGAGGGAGGGGAGACTGCTAAGTCTAAGAAGTTTCCTTCCTATACAGCCACCTACCAGTTCTGA |
