# Supplementary figures and images for: Reverse vaccinology approaches to design a potent multiepitope vaccine against the HIV whole genome: immunoinformatic, bioinformatics, and molecular dynamics approaches
Source: BMC Infect Dis. 2024 Aug 28;24:873. doi: 10.1186/s12879-024-09775-2 (PMC11360854; doi:10.1186/s12879-024-09775-2)

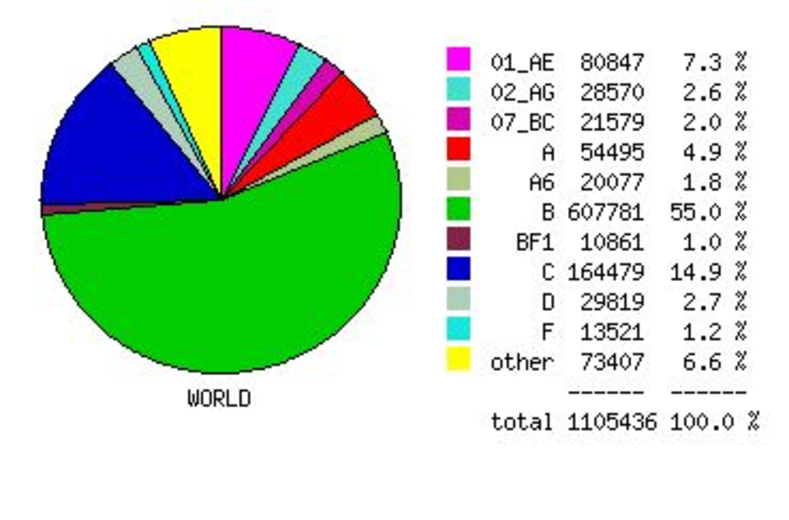

Supplement: Supplementary file 2 — Supplementary Material 2 [file 12879_2024_9775_MOESM2_ESM.tif]

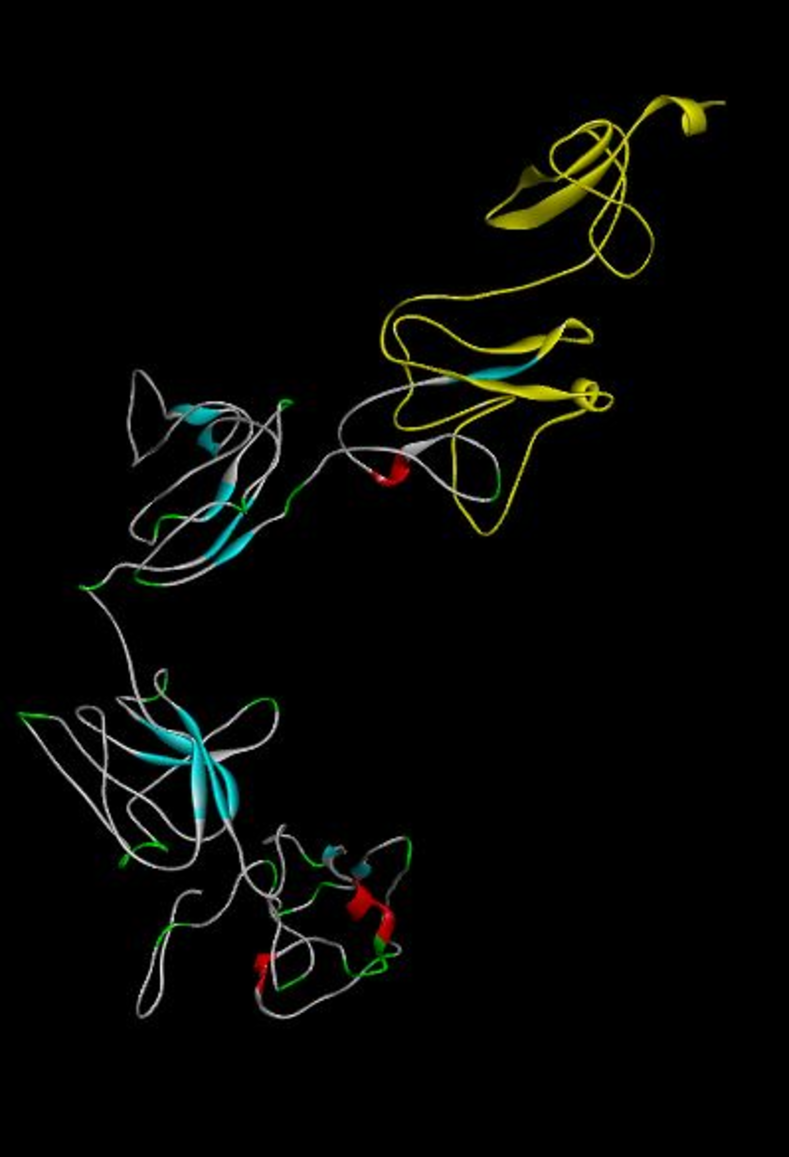

Supplement: Supplementary file 3 — Supplementary Material 3 [file 12879_2024_9775_MOESM3_ESM.tif]

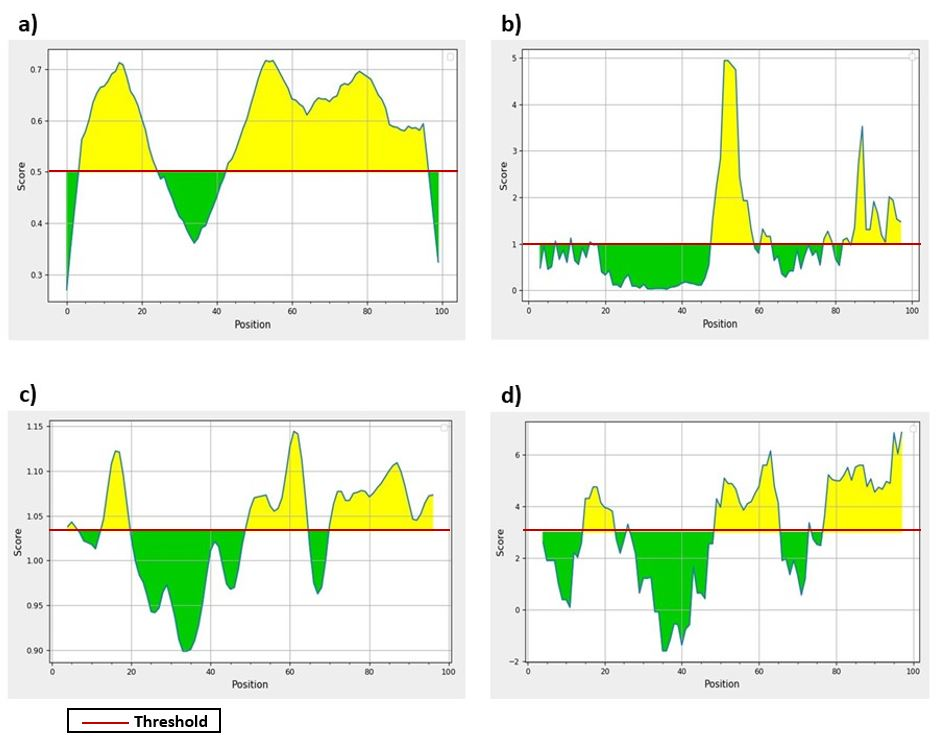

Supplement: Supplementary file 4 — Supplementary Material 4 [file 12879_2024_9775_MOESM4_ESM.tif]

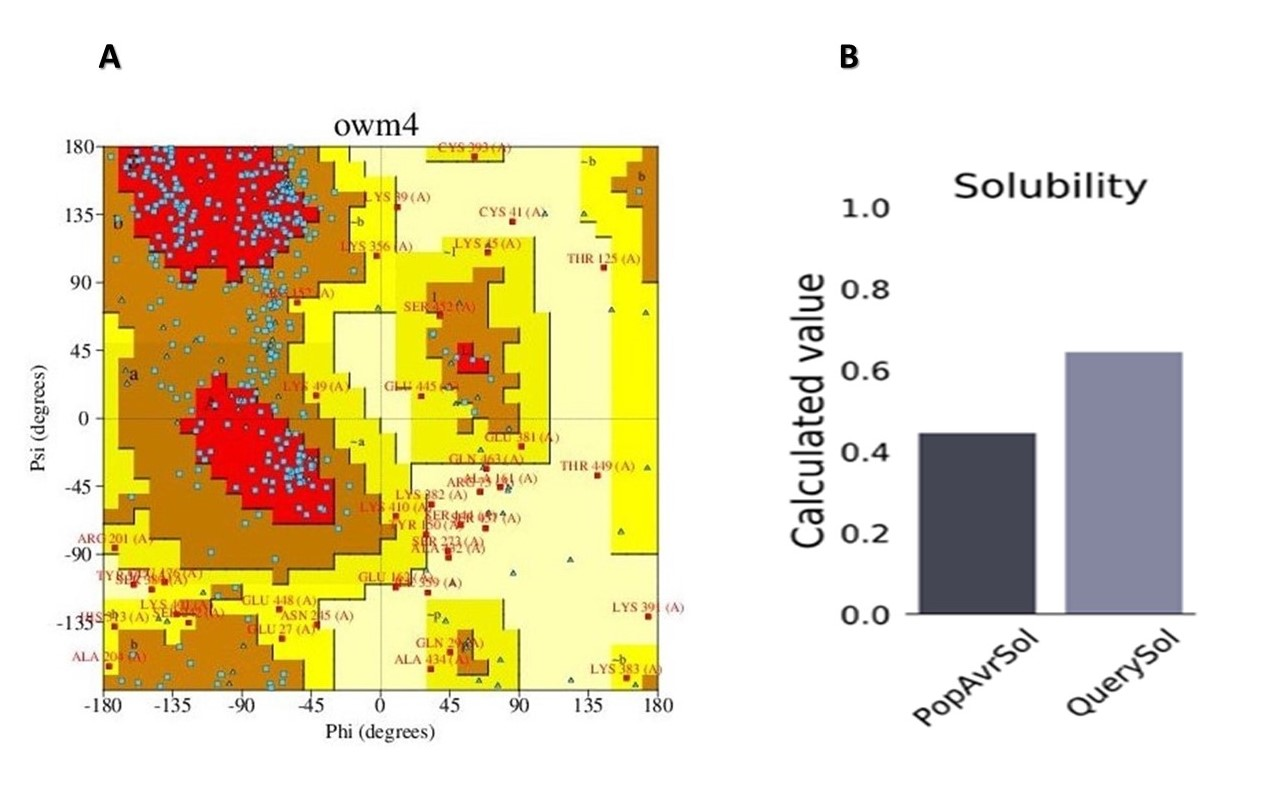

Supplement: Supplementary file 5 — Supplementary Material 5 [file 12879_2024_9775_MOESM5_ESM.tif]

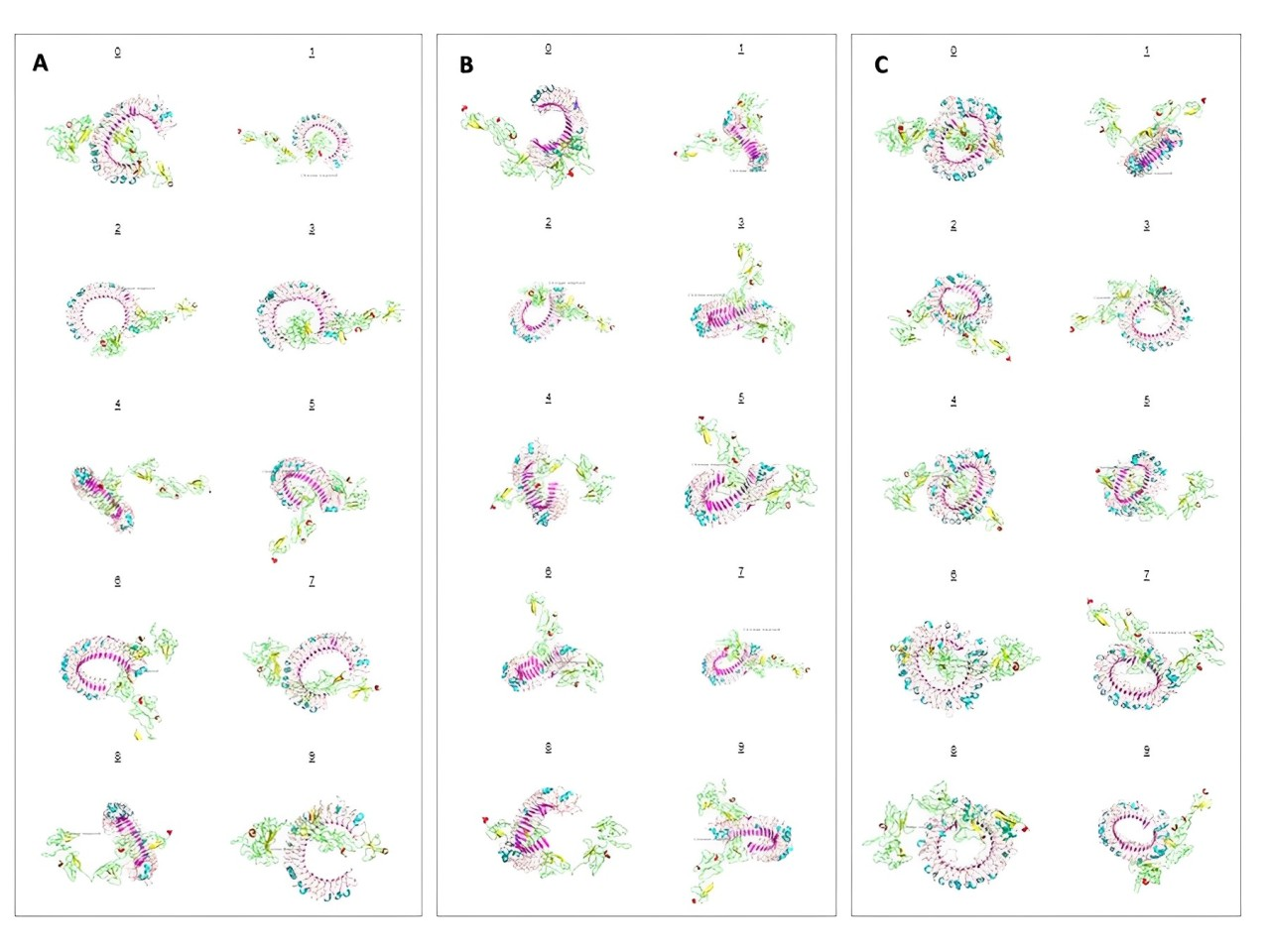

Supplement: Supplementary file 6 — Supplementary Material 6 [file 12879_2024_9775_MOESM6_ESM.tif]

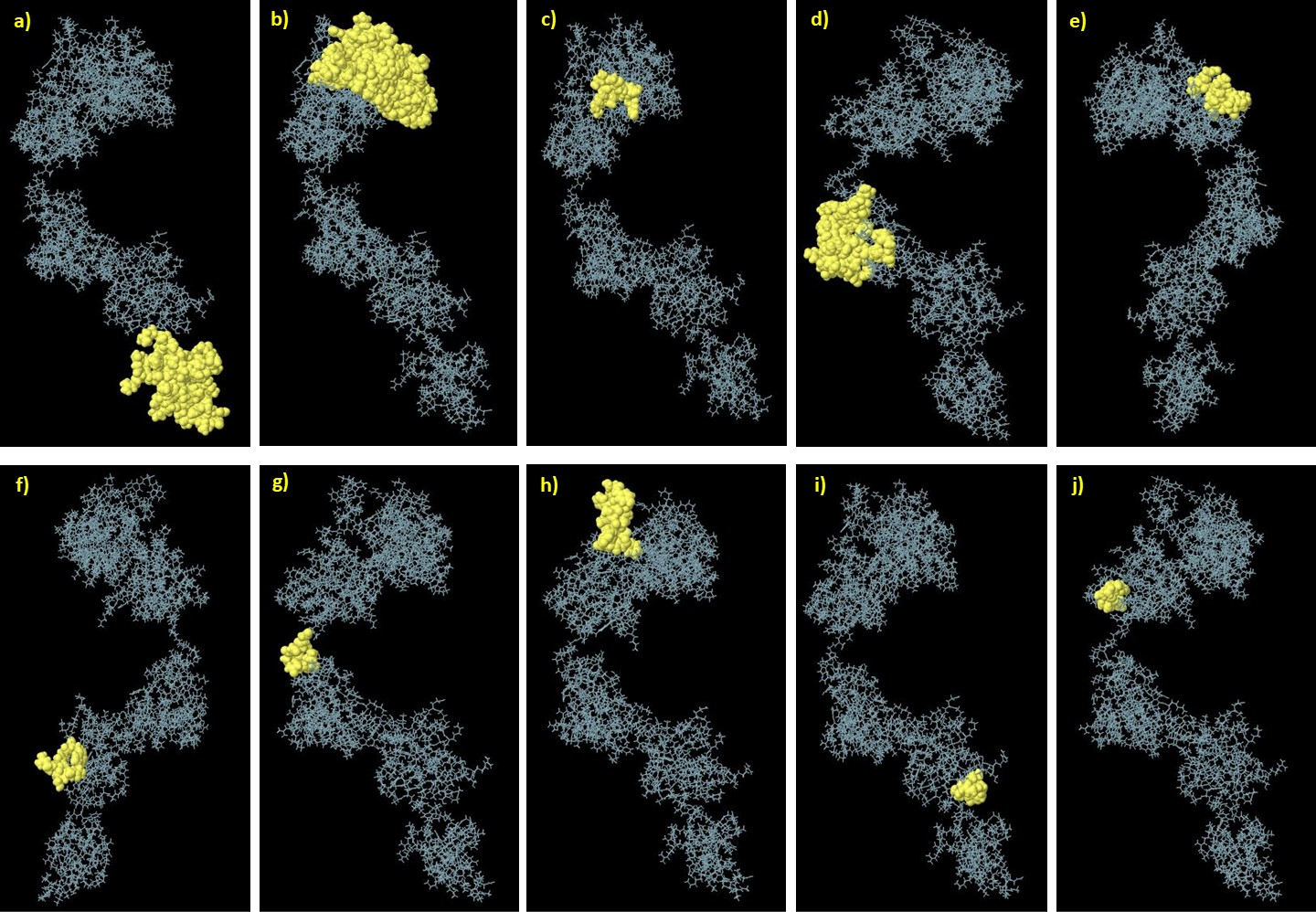

Supplement: Supplementary file 7 — Supplementary Material 7 [file 12879_2024_9775_MOESM7_ESM.tif]

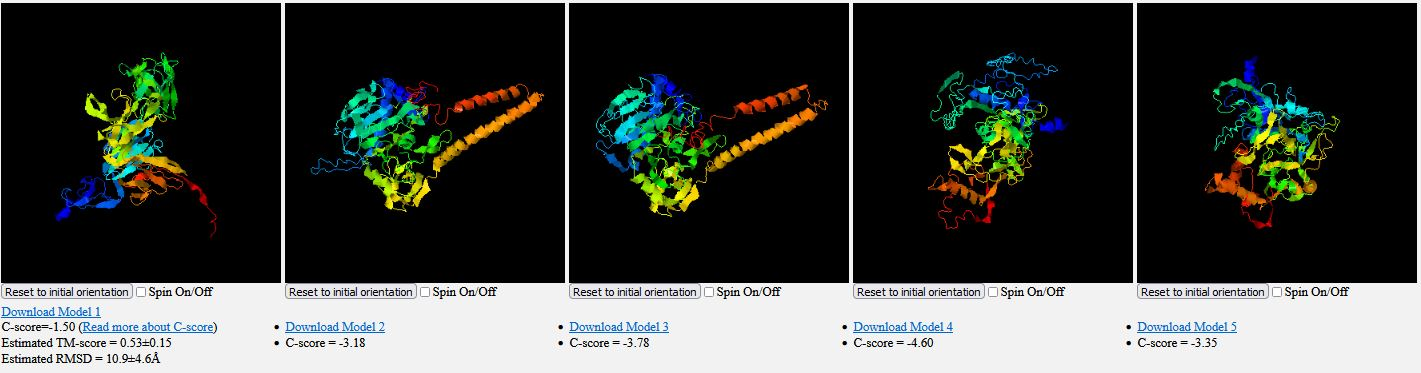

Supplement: Supplementary file 8 — Supplementary Material 8 [file 12879_2024_9775_MOESM8_ESM.tif]
